# The Coordination of Cell Growth during Fission Yeast Mating Requires Ras1-GTP Hydrolysis

**DOI:** 10.1371/journal.pone.0077487

**Published:** 2013-10-16

**Authors:** Cathryn Weston, Michael Bond, Wayne Croft, Graham Ladds

**Affiliations:** Division of Biomedical Cell Biology, Warwick Medical School, University of Warwick, Coventry, United Kingdom; University of Cambridge, United Kingdom

## Abstract

The spatial and temporal control of polarity is fundamental to the survival of all organisms. Cells define their polarity using highly conserved mechanisms that frequently rely upon the action of small GTPases, such as Ras and Cdc42. *Schizosaccharomyces pombe* is an ideal system with which to study the control of cell polarity since it grows from defined tips using Cdc42-mediated actin remodeling. Here we have investigated the importance of Ras1-GTPase activity for the coordination of polarized cell growth during fission yeast mating. Following pheromone stimulation, Ras1 regulates both a MAPK cascade and the activity of Cdc42 to enable uni-directional cell growth towards a potential mating partner. Like all GTPases, when bound to GTP, Ras1 adopts an active conformation returning to an inactive state upon GTP-hydrolysis, a process accelerated through interaction with negative regulators such as GAPs. Here we show that, at low levels of pheromone stimulation, loss of negative regulation of Ras1 increases signal transduction via the MAPK cascade. However, at the higher concentrations observed during mating, hyperactive Ras1 mutations promote cell death. We demonstrate that these cells die due to their failure to coordinate active Cdc42 into a single growth zone resulting in disorganized actin deposition and unsustainable elongation from multiple tips. These results provide a striking demonstration that the deactivation stage of Ras signaling is fundamentally important in modulating cell polarity.

## Introduction

The ability of cells to maintain their shape and polarity during growth is an essential prerequisite of life. The mechanisms by which cells achieve this are highly conserved, relying on nucleation and growth of actin filaments to re-organize their cytoskeleton in response to changes in their environment. In many cells the regulation of small GTPases is fundamental to the control of polarized growth. Small GTPases act as molecular switches with an active GTP-bound form that interacts with downstream effector proteins and an inactive GDP state. They exhibit intrinsic GTPase activity that hydrolyses GTP to GDP leading to deactivation, but this rate is slow and is enhanced via interaction with GTPase-activating proteins (GAPs). Once GDP-bound, reactivation occurs through the action of guanine nucleotide-exchange factors (GEFs) that catalyze the release of GDP, allowing the more cellular abundant GTP to bind. 

**Figure 1:**
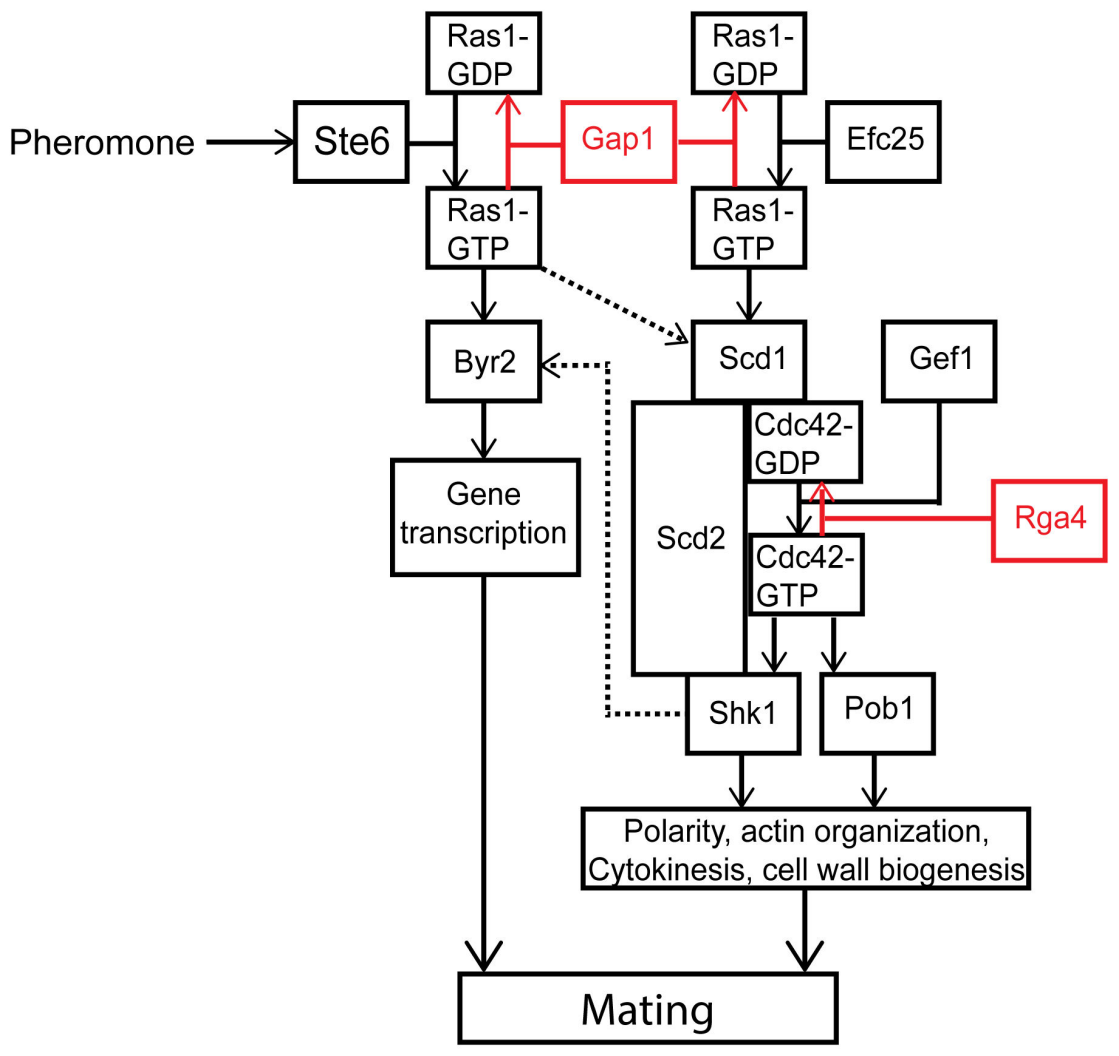
Schematic representation of Ras1 signaling cascades. Ras1 signals via two distinct effectors to control of both pheromone-inducible and routine cell growth pathways. In the absence of pheromone the GEF, Efc25 promotes GDP for GTP exchange on Ras1. Through a complex with a Rho-GEF, Scd1, Ras1-GTP activates the essential G protein, Cdc42 so allowing polarized cell growth, faithful chromosome segregation and cell division. A second GEF for Cdc42, Gef1, is required to initiate bipolar growth but is not activated by Ras1. Upon pheromone stimulation, Ras1 activation is increased through interaction with another Ras1-GEF, Ste6. This pool of Ras1-GTP propagates the transcriptional response (acting via a second effector, Byr2 and a MAPK cascade). Pheromone-activated Ras1 also induces further GTP exchange on Cdc42 through activation of Scd1 promoting unidirectional cell growth towards a mating partner. Ras1 and Cdc42 contain a slow intrinsic ability to hydrolyze GTP, but the reaction is accelerated through interaction with GAPs, Gap1 and Rga4 (shown in red). Cross-talk between the two pathways is highlighted with dashed arrows.

In all eukaryotic cells small GTPases play an important role in the establishment and maintenance of cell polarity with highly conserved signaling cascades identified from yeast to mammalian cells. Therefore, simpler, unicellular systems such as the fission yeast, *Schizosacharomyces pombe* have long been used to investigate the control of cell polarity [1]. *S. pombe* displays a characteristic rod-shaped morphology growing by polarized tip extension. This polar growth is regulated by progression through the cell cycle and is also influenced by external cues such as nutrient limitation and the presence of mating pheromones [2]. Importantly, fission yeast polarity is controlled by the actions of two conserved GTPases, Cdc42 and Ras1 [3] (Figure 1).

Cdc42, a Rho-GTPase homologue, is required to establish growth zones via actin nucleation, a mechanism highly conserved among eukaryotic cells. Studies in both mammalian systems [4] and fission yeast [5,6] have revealed a significant role for both the positive and negative regulation of Cdc42 during polarized cell growth. In *S. pombe* two GEFs (Scd1 and Gef1) and one GAP (Rga4) have been identified for Cdc42 which are spatially and temporally regulated to generate a gradient of activity ensuring growth occurs only at the cell poles [7]. Gef1 and Scd1 share the essential role of Cdc42 activation and a double deletion is not viable however, the two proteins are not functionally redundant. Gef1 regulates the temporally controlled transition from monopolar to bipolar growth ensuring the correct cell size is achieved before cell division [8]. Scd1 is required to concentrate Cdc42 activity at the cell tip focusing growth at this location. Deletion of Scd1 or its activator, a second small GTPase, Ras1 results in a complete loss in polarity [9] since Cdc42 activity is not as efficiently directed to the cell tip and as such growth occurs over a wider area resulting in round cells [7]. 

In addition to their role in regulating mitotic cell growth, Scd1 and Ras1 are also required for mating. Upon nutrient limitation *S. pombe* cells undergo sexual differentiation resulting in an arrest in G_1_ and the expression of mating pheromones and their receptors [2]. Many of these responses are controlled by the transcription factor Ste11, which is regulated through phosphorylation by members of a mitogen-activated protein kinase (MAPK) cascade that is stimulated by Ras1 [10]. Importantly, Ras1 also provides the link between pheromone stimulation and activation of the Scd1-Cdc42 pathway required for polarized (uni-directional) cell growth during the mating response (termed shmoo).

Ras1 activity requires similar regulation to Cdc42 having two temporally restricted GEFs; Efc25 for mitotic growth [11] and Ste6, induced upon nitrogen starvation to regulate mating responses [12]. A single predicted GAP for Ras1, Gap1 has been identified [13]. Deletion of Gap1 or expression of hyperactive Ras1 mutants (analogous to the GTPase defective, oncogenic transitions observed in human cancer cells) results in cells that are hypersensitive to pheromone stimulation but sterile [13]. Interestingly there is no effect on the polar morphology of mitotically growing cells containing the same hyperactive Ras1 mutations. Given these contrasting observations for the requirement of negative regulation, we sought to quantitatively investigate the role of Ras1-GTP-hydrolysis on signal propagation. 

We confirm, using Förster resonance energy transfer (FRET) that Gap1 interacts with Ras1-GTP. Furthermore, we identify a difference between the mechanisms Ras1 uses to mediate signal propagation. We demonstrate that, whilst hyperactivating mutations, predicted to prolong Ras1 activation, increase signal transduction via the MAPK cascade, a reduction in signal propagation via Cdc42 was observed. Significantly, we find that these cells are unable to initiate polarized cell growth in response to mating pheromone. Cells appear unable to coordinate the localization of Cdc42 and therefore fail to establish a single growth zone. Pheromone stimulation of these cells results in attempted growth from multiple sites and ultimately cell death. These results offer a novel insight into how Ras proteins regulate cell polarity and highlight an essential role for negative regulation of G protein activation to ensure efficient propagation via downstream signaling cascades.

## Results

### Gap1 is recruited to Ras1 in a GTP-dependent manner

Genetic analysis suggests that Gap1 acts primarily as a negative regulator of Ras1 [13] however; it has not been directly demonstrated to interact with Ras1-GTP *in vivo*. We expressed CFP-Ras1 and Gap1-YFP fusion constructs in a fission yeast strain (JY1618) lacking endogenous copies of both the genes. This strain also lacks Cyr1 generating a genotype mimicking nutrient limitation thereby allowing pheromone stimulation but producing cells of a smaller size when grown in minimal media [14-16]. Protein function was unaffected by expression from inducible plasmids or incorporation of the fluorescent moiety (Figure S1).

**Figure 2:**
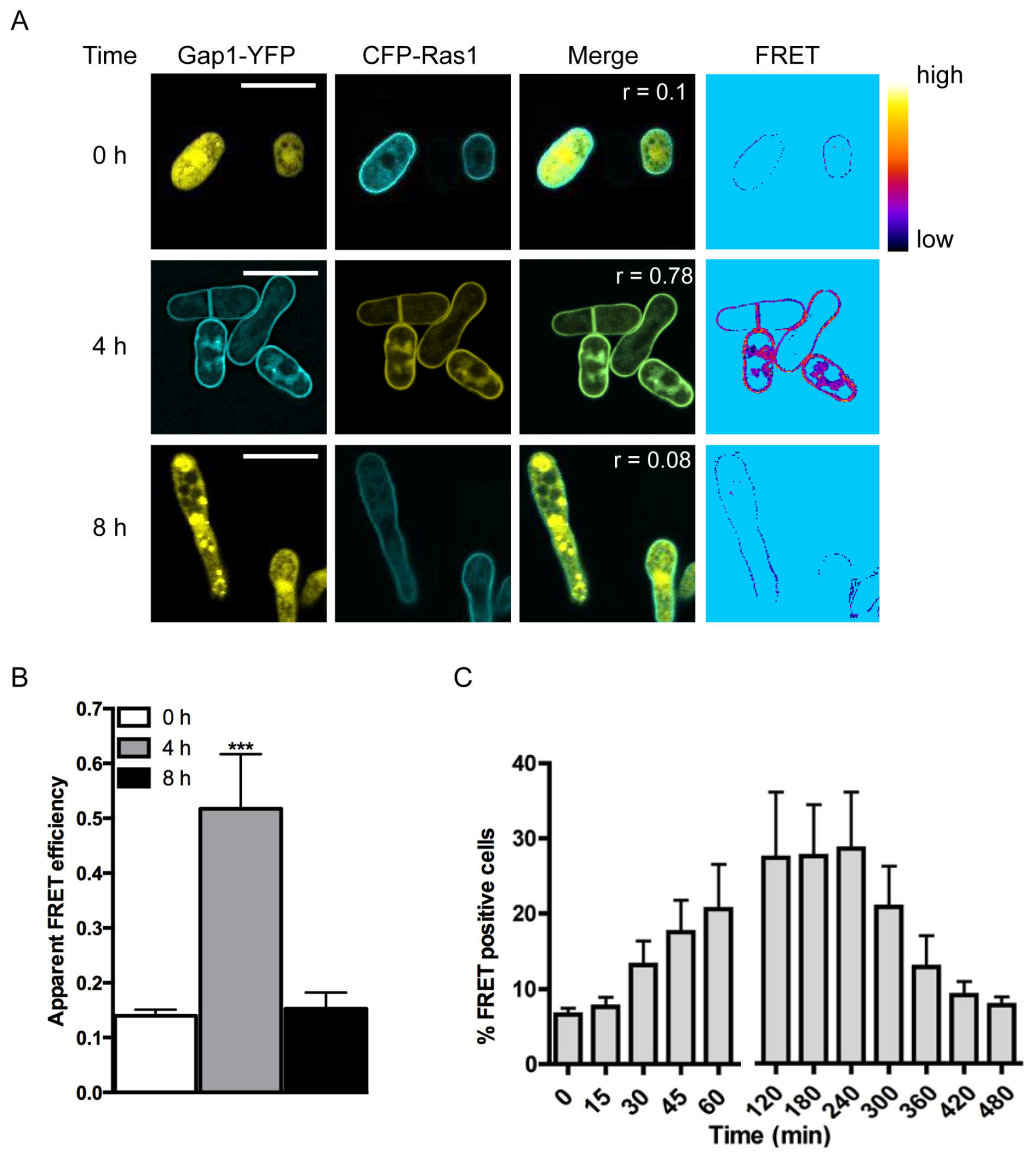
Gap1 interacts with Ras1-GTP following pheromone stimulation. **A**, *Δras1Δgap1* cells transformed with Gap1-YFP and CFP-Ras1 were treated with 10 μM pheromone and imaged to determine localization of the proteins and confirm an interaction using FRET. Pearson’s correlation coefficients (r) and regions of FRET were determined using the Image J FRET and colocalization plugin. Heat-map shows FRET efficiency low (black) to high (yellow). **B**, Quantified apparent FRET efficiency of cells as indicated, determined using FRET-SE (displayed as mean (±SEM) of 10 cells analyzed). FRET efficiency was significantly elevated after treatment with 10 μM pheromone. Statistical significance determined using a two-tailed Students’s t test; *** representing p < 0.001. **C**, FACS-based FRET analysis of cells described in A. Data shown are mean values (±SEM) of five independent experiments.

Consistent with previous observations [17], CFP-Ras1 localized to plasma and endomembrane structures (Figure 2A). In contrast, Gap1-YFP appeared diffuse throughout the cytosol and nucleus. Following 4 h stimulation with pheromone, Gap1-YFP was observed to translocate to the plasma membrane colocalizing with CFP-Ras1 (Pearson’s correlation coefficient (r) = 0.78) suggesting an interaction with Ras1, which was confirmed using FRET by sensitized-emission. Apparent FRET efficiencies were calculated for individual cells 0, 4 and 8 h following stimulation with 10 μM pheromone. A significant increase in FRET signal was observed 4 h post stimulation which returned to basal levels within 8 h (Figure 2B). To observe the temporal dynamics of the Gap1-YFP-CFP-Ras1 interaction we used fluorescence activate cell sorting (FACS, Figure S1F) to quantify the occurrence of FRET (Figure 2C). A small number (8 ± 0.1%) of mitotically growing cells (Time = 0, Figure 2C) co-expressing CFP-Ras1 and Gap1-YFP produced a FRET signal; this percentage increased 2-fold following 30 min pheromone stimulation, peaking at 2-4 h before returning to basal by 8 h. These dynamics are in broad agreement with that of the activation of Ras proteins determined for other organisms [18] and demonstrate that in fission yeast, Gap1 interacts with GTP-bound, active Ras1.

**Figure 3:**
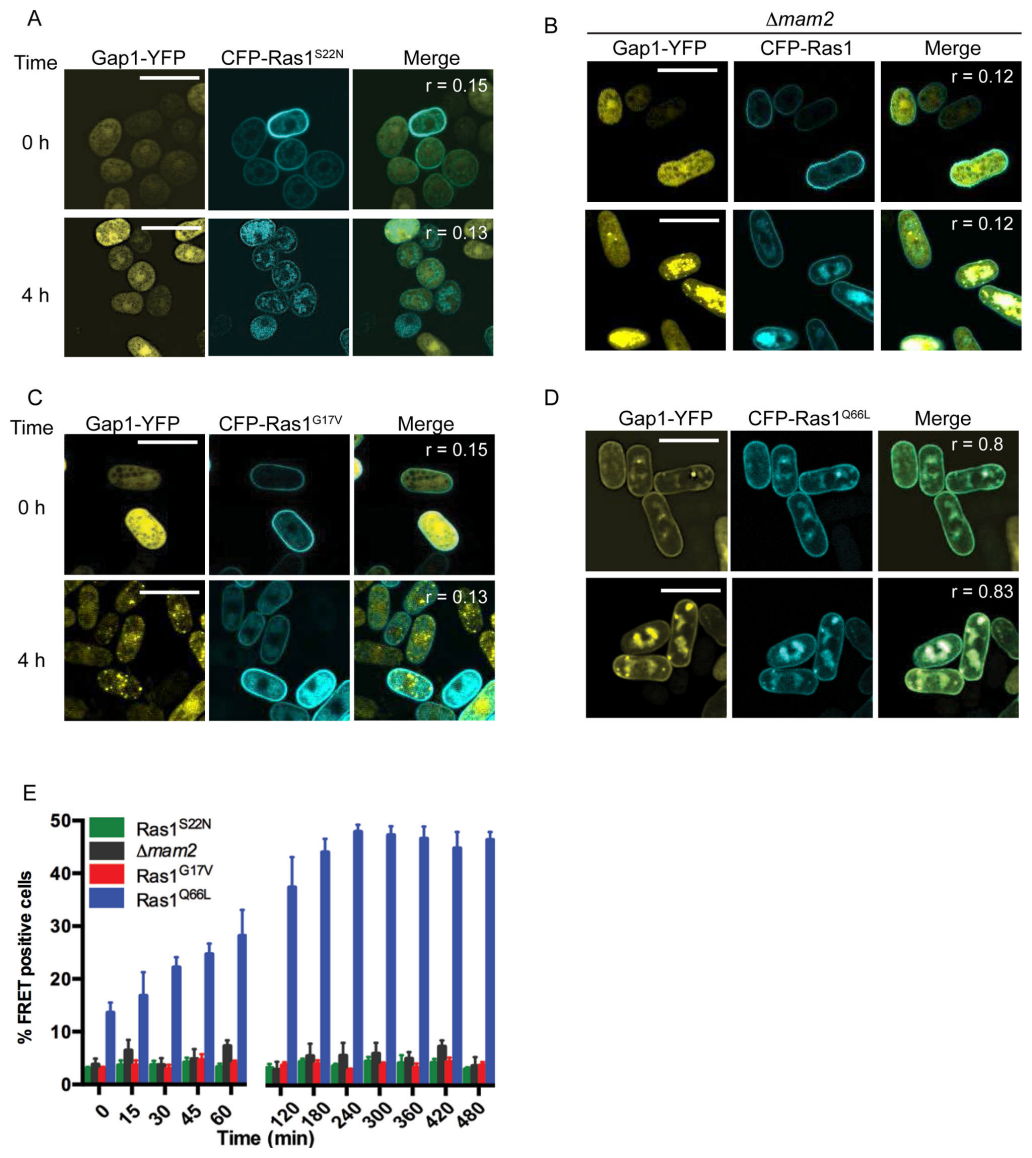
Gap1 requires pheromone-induced GTP-binding to interacts with Ras1-GTP. **A**-**D**, Fission yeast strains containing various CFP-Ras1 mutations and Gap1-YFP were treated with 10 μM pheromone and imaged to determine localization of the proteins. Pearson’s correlation coefficients (r) were determined using the Image J colocalization plugin. **A**, *Δras1Δgap1* cells transformed with Gap1-YFP and CFP-Ras1^S22N^, **B**, *Δras1Δgap1Δmam2* cells containing Gap1-YFP and CFP-Ras1, **C** *Δras1Δgap1* cells transformed with Gap1-YFP and CFP-Ras1^G17V^ and **D**, *Δras1Δgap1* cells containing Gap1-YFP and CFP-Ras1^Q66L^. **E**, Strains in **A-D** were analyzed using FACS-based FRET to demonstrate protein-protein interactions. Data shown are mean values (±SEM) of five independent experiments.

The requirement for activation of Ras1 through binding of GTP was confirmed using previously characterized Ras1 mutants engineered to contain a CFP-label. Expression of CFP-Ras1^S22N^ (the mammalian equivalent, Ras^S17N^ is unable to bind GTP [19]) prevented pheromone-induced translocation of Gap1-YFP to the plasma membrane (Figure 3A). Similarly, no change in Gap1-YFP localization (Figure 3B) was observed in a strain lacking the pheromone receptor, Mam2 [20]. These results demonstrate the requirement for GDP for GTP exchange, which is enhanced through pheromone stimulation, to promote interaction between CFP-Ras1 and Gap1-YFP.

Several hyperactivating mutations of human Ras proteins have been described. The Ras^G12V^ transition is predicted to prevent an interaction with GAPs [21]. Expression of the analogous CFP-Ras1^G17V^ in the fission yeast strains prevented translocation of Gap1-YFP to the plasma membrane (Figure 3C) and no increase in FRET signal was detected following pheromone stimulation (Figure 3E). This data is consistent with the notion that the Gly to Val transition in the P-loop of Ras proteins results in a reduced affinity for the GAP [21] and demonstrates that Gap1-YFP requires an interaction with CFP-Ras1 to translocate to the plasma membrane in *S. pombe*.

In contrast, the hyperactive Ras1^Q66L^ mutation (the mammalian equivalent, Ras^Q61L^, has been shown to prevent GTP hydrolysis) promoted Gap1-YFP association with the plasma and endomembrane structures (Figure 3D, r = 0.8). Pheromone stimulation resulted in an increase in the percentage of cells co-expressing CFP-Ras1^Q66L^ and Gap1-YFP generating a FRET signal (Figure 3E). Furthermore, the Gln66Leu mutation prevented a return to basal levels. These data support the hypothesis that this mutation does not prevent GAP-binding but reduces GTP hydrolysis [21] and further highlight that Gap1 interacts with Ras1 in a GTP-dependent manner in fission yeast. 

Gap1 had been predicted to accelerate the hydrolysis of GTP on Ras1 [13]. The dynamics of the CFP-Ras1 and CFP-Ras1^Q66L^ FRET interactions with Gap1-YFP strongly support this prediction: the incorporation of the Gln66Leu transition, which prevents GTP-hydrolysis, results in a sustained FRET signal, indicating that Gap1-YFP is not released until Ras1 becomes GDP-bound. 

### Gap1-mediated GTP hydrolysis is required for an efficient mating response

**Figure 4:**
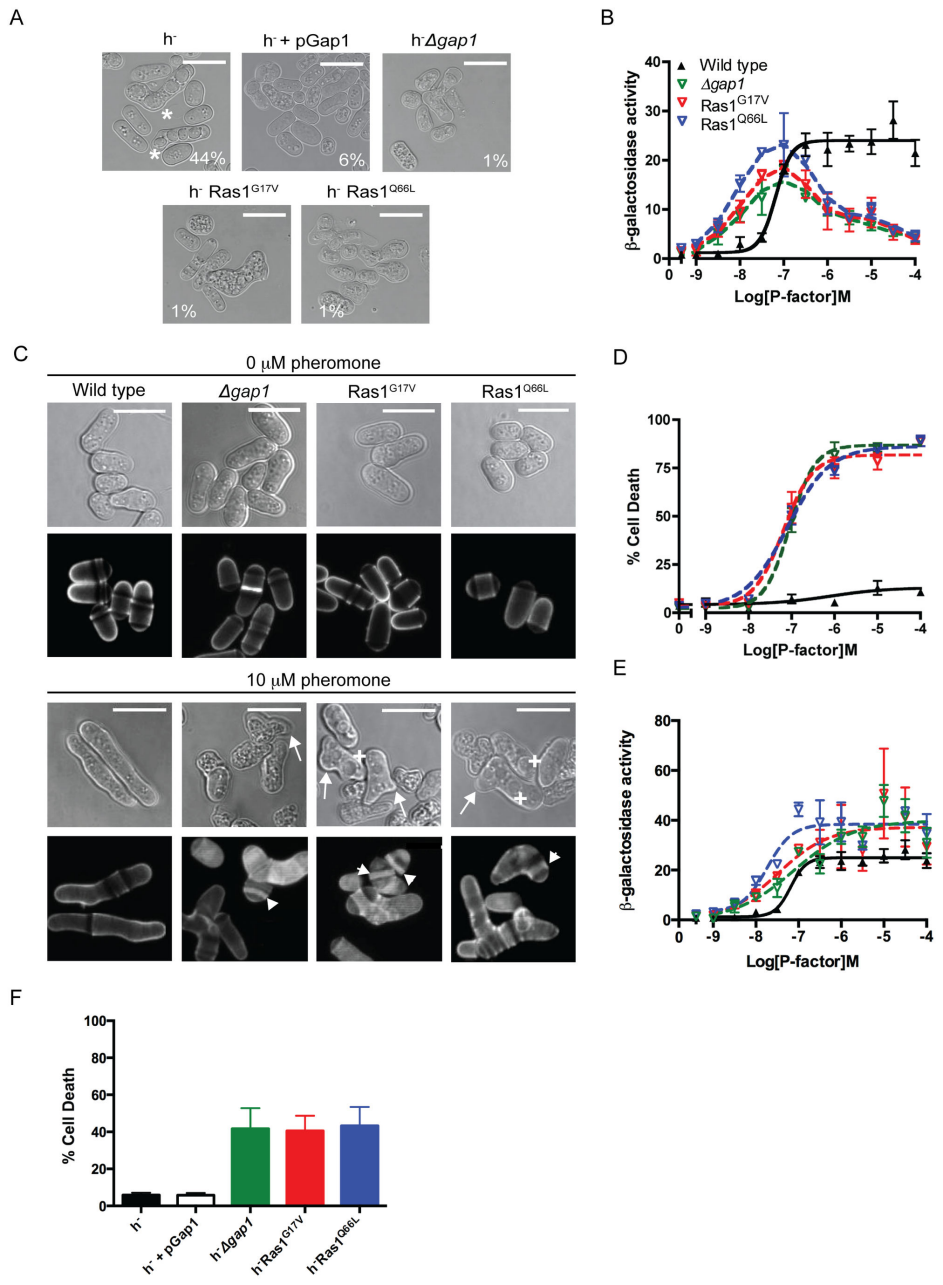
Ras1-GTP hydrolysis is essential during mating. **A**, Populations of *S. pombe* (h^+^ and h^-^) cells were imaged and scored for sporulation (asterix). Scale bars 10 μm. Values shown are % mated cells within each population. **B**, Pheromone-dependent transcription as determined using the *sxa2>lacZ* reporter for strains indicated. Ras1 GTPase-deficient strains display an initial increase in sensitivity to pheromone (< 0.1 μM) but signal is reduced at saturating concentrations (> 0.1 μM). **C**, Bright field and calcofluor staining in indicated genotypes. Upon pheromone exposure, wild type cells elongate from a single tip however, Ras1 GTPase-deficient strains display multiple elongation tubes (arrows), enlarged vacuoles (cross) and increased deposits of cell wall material (arrowheads). **D**, Pheromone-dependent changes in cell viability. Unlike wild type cells, Ras1-GTPase-defective strains display dose-dependent increase in cell death. **E**, Pheromone-dependent reporter gene activity from **B**, corrected for the number of viable cells within the population as measured in **D**. **F**, Percentage cell death within mating mixes shown in **A**. Values are mean of triplicate determinations (± standard error from the mean (SEM)).

Ras1-mediated signal transduction is required for fission yeast mating [22]. Increasing the cellular concentration of Gap1 (using the thiamine repressible *nmt1* promoter) resulted in cells that displayed a reduced mating efficiency (Figure 4A and Table S1). These results, combined with the observed decrease in the FRET signal (a marker for active Ras1) 4 h post pheromone stimulation (Figure 2C) suggest that the removal of Gap1 would prolong the active Ras1-state and therefore increase signal transduction and mating efficiency. However, counter-intuitively, deletion of *gap1* reduced mating efficiency (Figure 4A, [13]). Similar data was obtained for *S. pombe* cells expressing either the hyperactive Ras1^G17V^ or Ras1^Q66L^ mutants suggesting that loss in mating in *Δgap1* strains results from the absence of Gap1-mediated Ras1-GTP hydrolysis. These data indicate that prolonging the GTP-bound ‘active’ state of Ras1 paradoxically reduces signal transduction. 

To quantitatively investigate the molecular mechanisms underlying the reduced mating efficiency, we generated pheromone-responsive reporter strains either lacking *gap1* or where the endogenous *ras1* gene was replaced with the hyperactive Ras1 mutations. These previously described reporter strains contain the bacterial gene *lacZ*, at the *sxa2* locus [23,24]. Sxa2 is a carboxypeptidase that is only expressed following pheromone stimulation and Ras1-mediated activation of the MAPK cascade [25,26]. Therefore, expressing *lacZ* under the transcriptional control of the *sxa2* promoter provides a quantitative readout for Ras1-Byr2-mediated signal transduction, which is required for a mating response. All strains lacking *gap1* or containing a hyperactivating Ras1 mutation revealed an initial increase in pheromone sensitivity (<0.1 μM) however, when exposed to pheromone concentrations exceeding 0.1 μM, signaling was reduced 5-fold (Figure 4B). These observations are in contrast to the expected outcome from the removal of a negative regulator and may explain the loss in mating efficiency. 

### Gap1 is essential during the pheromone response

In addition to the activation of Byr2 for gene transcription, efficient mating in *S. pombe* requires the initiation of uni-directional growth to form a conjugation tube or shmoo (Figure 1) [2]. This polarized growth requires Ras1 activation of Scd1 [9] and can be studied using cells lacking Sxa2. Upon prolonged exposure to 10 μM pheromone (16 h) *Δsxa2* strains exhibit abnormally long conjugation tubes generated from a single tip [27] (Figure 4C). Under the same treatment conditions our hyperactive mutant strains failed to exhibit a defined polarity, often (approximately 33%, n = 103 cells) attempting to elongate from multiple sites (Figure 4C, arrows). In addition, many cells displayed enlarged vacuoles (Figure 4C, cross) and a number of cellular fragments were also observed. 

Staining with calcofluor white, a marker for cell wall material revealed that many cells within the mutant populations contained a build up of cell wall material within their cytosol (Figure 4C, arrow heads) appearing to have died post-pheromone treatment. To confirm this observation, we used a commercially available cell viability assay (LIVE/DEAD^®^ *Funga* light^TM^ yeast viability kit (Invitrogen Ltd)) to quantify the percentage of cell death within a population following pheromone stimulation (Figure 4D). All mutant strains displayed a dose-dependent increase in percentage cell death, reaching a maximum at 1 μM pheromone. The pheromone-dependent reduction in the number of viable cells corresponded to the loss in transcriptional reporter gene activity observed in the mutant strains. Correcting the transcriptional reporter gene activity to account for the loss in cell viability revealed an increase in maximal response in the hyperactive mutant strains compared to wild type cells (Figure 4E). These data are consistent with observations for human oncogenic Ras mutations, which increase signal transduction via the Byr2 homologue, Raf1. These data suggest that the decrease in reporter gene activity observed at high (>0.1 μM) pheromone concentrations in the absence of Ras1-GTP hydrolysis results from a significant reduction in viable cells and not a loss in Ras1-mediated activation of Byr2. Similar defects in cell morphology and a loss in cell viability (Figure 4F) were observed in the mating mixes of strains lacking *gap1* or containing hyperactivating Ras1 mutations demonstrating that Gap1-accelerated Ras1-GTP hydrolysis is essential at the physiological concentrations of pheromone required for mating.

### Gap1-mediated Ras1-GTP hydrolysis is required during cell elongation

**Figure 5:**
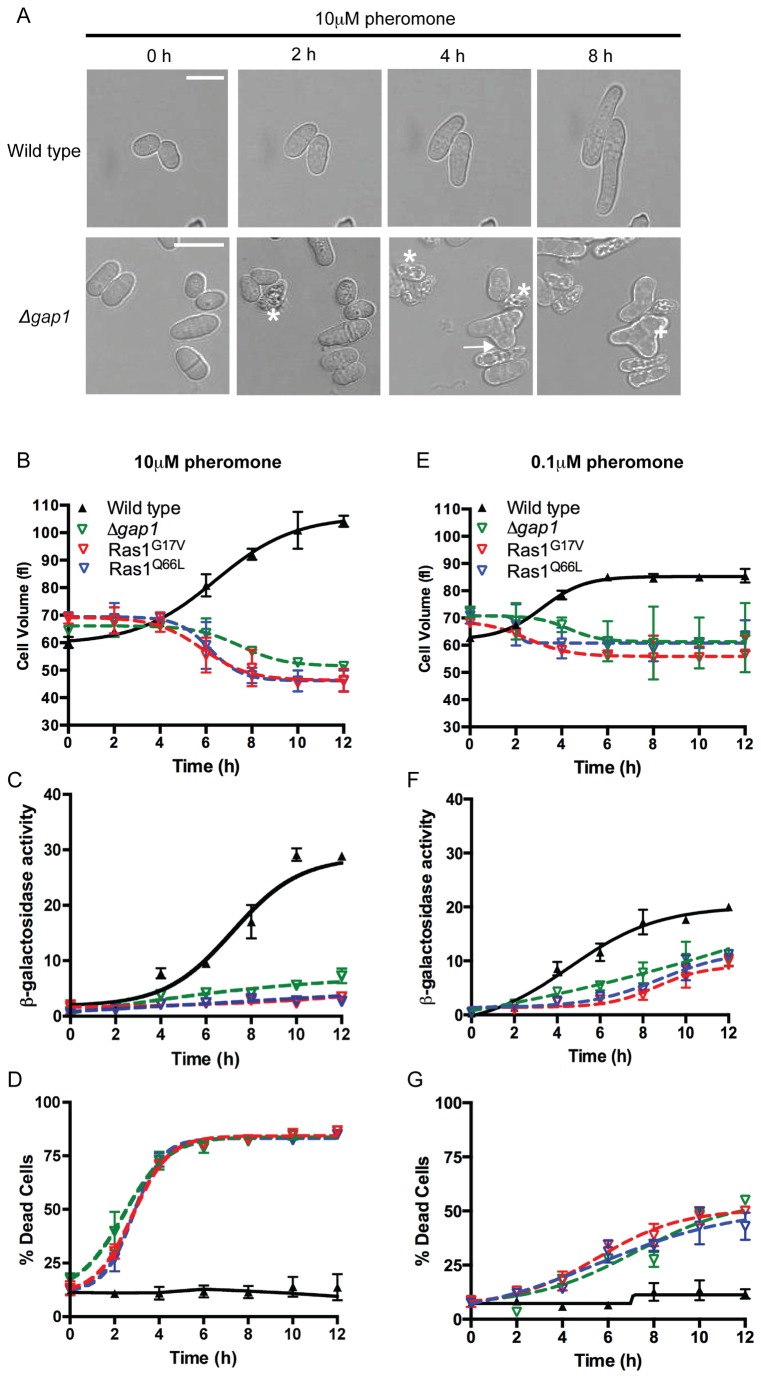
Ras1-GTP-Gap1 interaction is essential during pheromone-directed cell elongation. **A**, Images of morphological changes upon 10 μM pheromone stimulation. Wild type cells elongate from a single tip, in contrast, the presence of lysed cells (asterix), multiple projection tips (arrows) and enlarged vacuoles (cross) was noted in Ras1 GTPase-defective strains. Scale bar 10 μm. See also Movies S1-4. **B**-**D**, Quantification of pheromone-dependent changes in morphology, gene transcription and cell viability following exposure to 10 μM pheromone. **E**-**G**, Quantification of pheromone-dependent changes in morphology, gene transcription and cell viability following exposure to 0.1 μM pheromone. All data is mean (±SEM) of three individual experiments.

Having determined that Ras1-GTP hydrolysis is essential during fission yeast mating, we sought to elucidate the time during the response when signal termination becomes critical. Following stimulation with 10 μM pheromone, the *sxa2>lacZ* reporter strains elongate from a single tip forming a shmoo (Figure 5A and Movie S1). This change in morphology can be quantified as an increase in cell volume using a Coulter channelyzer [28], beginning 2–4 h post stimulation, reaching a maximum by 10–12 h (Figure 5B) with similar dynamics observed for transcriptional reporter gene activity (Figure 5C). Mutant strains exposed to 10 μM pheromone failed to establish a defined, single growth site during the same time-frame (Movies S2-4). Many cells within the population displayed multiple projection tips (Figure 5A) after 4 hours coinciding with the time at which a significant increase in cell death was observed (Figure 5D). This cell death resulted in a decrease in cell volume as measured using the Coulter channelyzer since dead *S. pombe* cells appear small and rounded. Similarly, due to the large increase in dead cells within the population little reporter gene activity was observed. Significantly, the time at which the mutant cells begin to display aberrant cell morphologies and a rapid increase in cell death corresponds to the initiation of shmoo formation in the wild type strain. These observations suggest that Gap1-accelerated Ras1-GTP hydrolysis becomes essential during the activation of Scd1-directed polarized cell growth. 

To investigate the temporal dynamics of Ras1-mediated signal transduction in the mutant strains we used a sub-lethal concentration of pheromone (0.1 μM), observed to cause < 50% cell death within a population (Figure 4D). Wild type cells exposed to 0.1 μM pheromone for 12 h did not obtain the same maximal level in either the morphological or transcriptional reporter gene assays as those observed at the high pheromone concentration, although the dynamics were similar (Figure 5E-F). In the mutant population a small increase in transcriptional reporter gene activity was detected however, no significant increase in cell volume was observed. Notably a reduction, in pheromone-induced cell death in mutant strains treated with 0.1 μM pheromone for 12 h (Figure 5G) compared to 10 μM was observed. Previously it has been shown that gene transcription occurs at a lower concentration of pheromone than shmoo formation [6]. Therefore these data further indicate that Ras1-GTP hydrolysis is essential during pheromone-induced, polarized cell growth. The lower concentration of pheromone induces some gene transcription but this is insufficient to stimulate cell elongation thereby reducing the number of cells that die. Failure to terminate Ras1 signaling at the high concentrations of pheromone required for mating appears to inhibit formation of a single conjugation tube; instead the mutant cells attempt to elongate from multiple sites.

### Differential requirements for Ras1-GTP hydrolysis

**Figure 6:**
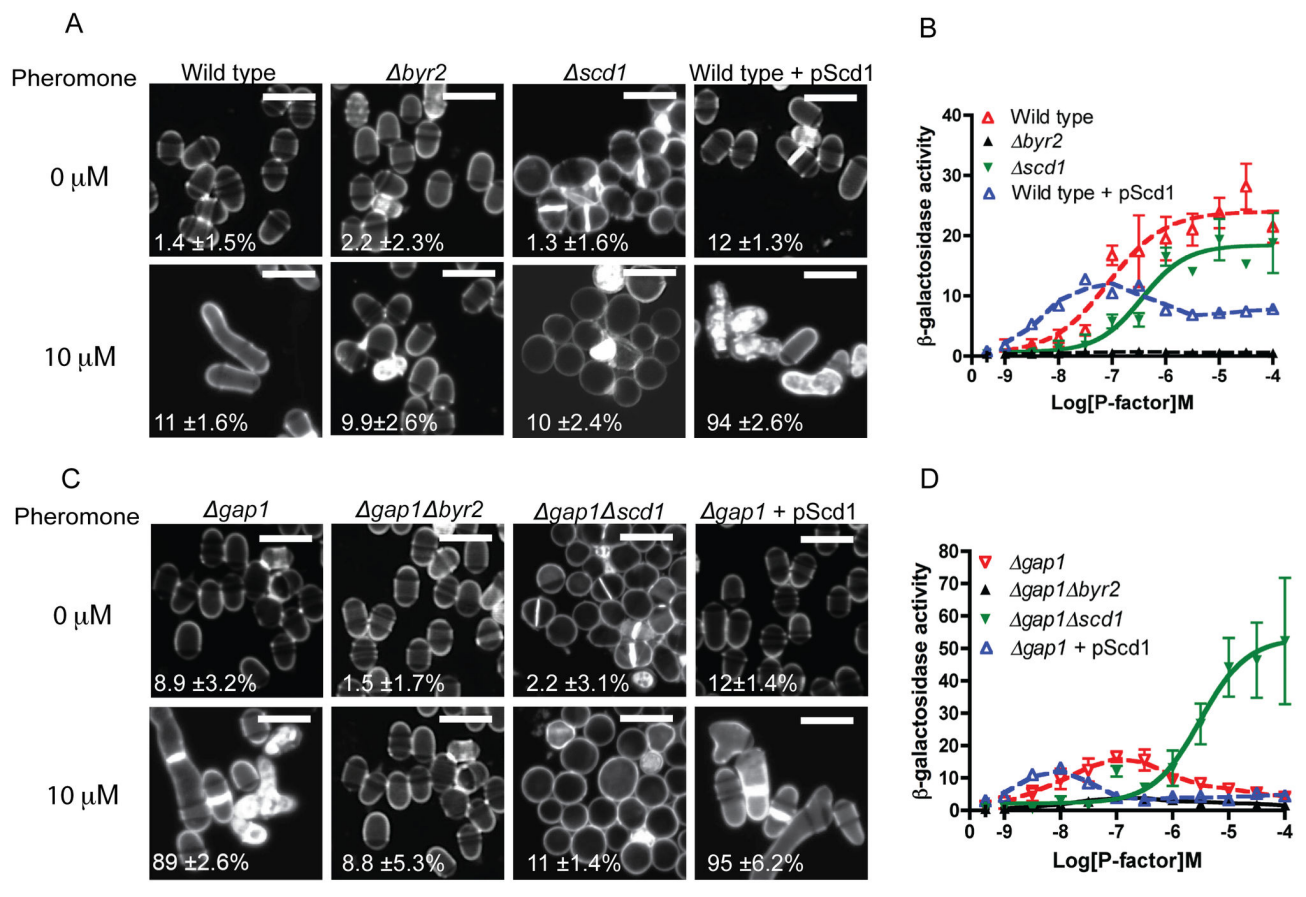
Ras1-GTP hydrolysis is essential to mediate cell polarity but not MAPK signaling. **A** and **C**, Calcofluor white staining of indicated strains treated with 10 μM pheromone. Scale bar 10 μm. Values shown are percentage loss of cell viability for each population. **B** and **D**, Pheromone-dependent transcription as determined using the *sxa2>lacZ* reporter for the stains indicated. Removal of Scd1 from cells lacking Gap1 prevented pheromone-induced cell death while enabling an elevated transcriptional response. All values are mean of triplicate determinations (±SEM).

For successful mating, Ras1 signal transduction proceeds via two distinct effectors, Byr2 and Scd1 (outlined in Figure 1). The above data indicates that activation of the Scd1 pathway, which regulates shmoo formation, in strains lacking Gap1 results in cell death, thereby reducing the reporter gene activity detected from Byr2 activity. To investigate if Ras1-GTP hydrolysis is essential for all downstream signaling or only that which occurs via Scd1, *S. pombe* strains lacking each effector were created. Deletion of Byr2 from wild type (Figure 6A-B) and *Δgap1* (Figure 6C-D) strains prohibited all pheromone-induced responses. In contrast, in the absence of Scd1, shmoo formation was inhibited whilst the maximal level of MAPK signaling was unaffected (Figure 6A-B). Significantly, deletion of Scd1 from the *Δgap1* strain prevented pheromone-induced cell death (Figure 6C) and enabled an increased transcriptional response (Figure 6D). 

Elevated expression of Scd1 (pScd1) resulted in increased sensitivity to pheromone in both wild type and *Δgap1* strains. Furthermore, expression of pScd1 in wild type cells reduced the maximal level of reporter gene activity, presumably as a result of increased cell death within the population (Table S2). From these data it appears that Ras1-GTPase activity is essential for efficient signal propagation via Scd1 but not Byr2 highlighting an interesting contrast between the requirements for Ras-GTP hydrolysis for signal propagation via the two distinct effectors.

### Pheromone-induced cell death results from a decrease in Cdc42 signal transduction

Scd1 is one of two GEFs for the Rho-like GTPase, Cdc42 [29] Cdc42 has a critical role in maintaining both ordered actin cable formation to allow maintenance of polar cell morphology and also in the regulation of efficient cell division [30]. We next sought to investigate how the hyperactivation of Ras1 affects the regulation of Cdc42 activity. 

**Figure 7:**
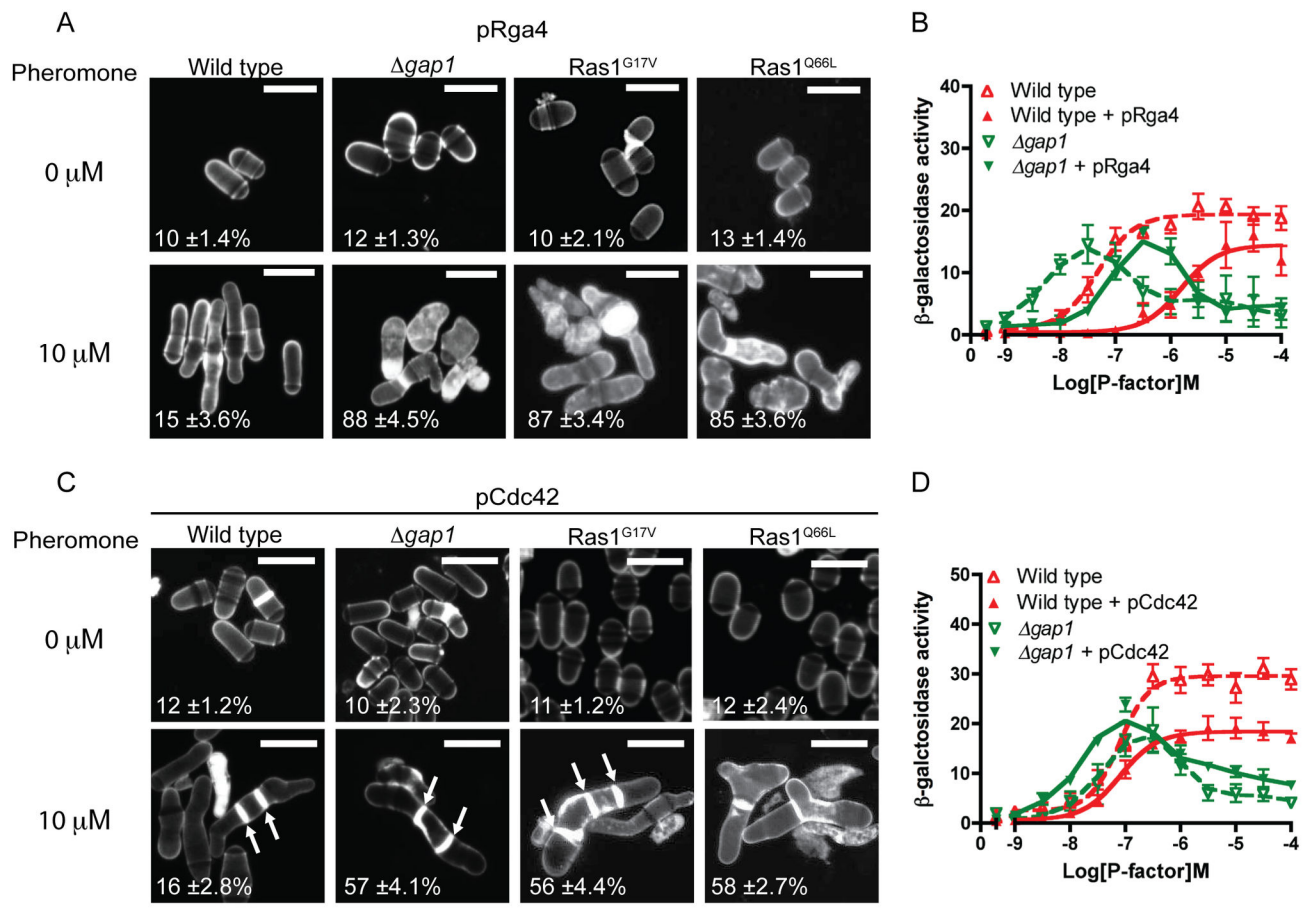
Pheromone-induced cell death is due to reduced Cdc42 signal transduction. **A**, **and** **C** Values shown are percentage loss of cell viability. Scale bar 10 μm **A**, Calcofluor white staining of cells from indicated genotypes treated with pheromone. **B**, Pheromone-dependent transcription was quantified using the *sxa2>lacZ* reporter construct in strains expressing pRga4. **C**, Cells transformed with Cdc42 (pCdc42) were treated with pheromone, imaged as for **A**, many cells displayed multiple septa (arrows). **D**, Transcriptional response to pheromone was determined and revealed a slight restoration of maximal signal in the *Δgap1* strain containing pCdc42. Values shown are mean of triplicate determinations (± SEM). Scale bar 10 μm.

Increased expression of Rga4 (a GAP for Cdc42) [31] did not prevent the pheromone-induced cell death in our mutant strains (Figure 7A and Table S2). In all strains analyzed, there was a reduction in pheromone-sensitivity upon increased expression of Rga4 (Figure 7B and Figure S2). This may arise from a decrease in Shk1 [32] activation (Figure 1), an essential Cdc42 effector, reported to enhance pheromone-induced transcriptional responses via interaction with Byr2 [33]. These data suggest that the reduction in cell viability observed in strains lacking Ras1-GTP hydrolysis does not result from an increase in Cdc42 activity. 

In contrast, increasing the cellular concentration of Cdc42 was able to partially restore mating efficiency (Table S1), morphological defects and increased the number of cells surviving pheromone treatment (Figure 7C and Table S2). However, signaling measured using the pheromone-induced transcriptional reporter was not fully restored (Figure 7D and Figure 2). This may be due to the requirement for both positive and negative regulation of Cdc42 activity, since increased expression of Cdc42 resulted in deregulation of septation (Figure 7C, arrows) and reduced the maximal reporter gene response (~2-fold) in wild type strains due to an increase in cell death (Table S2). These observations combined, indicate that the loss of cell viability and mating in strains containing hyperactive Ras1-mutations is due to reduced, not prolonged, Cdc42-effector activation.

### Reduced Ras1-GTP hydrolysis results in loss of actin coordination required for polarized cell growth

**Figure 8:**
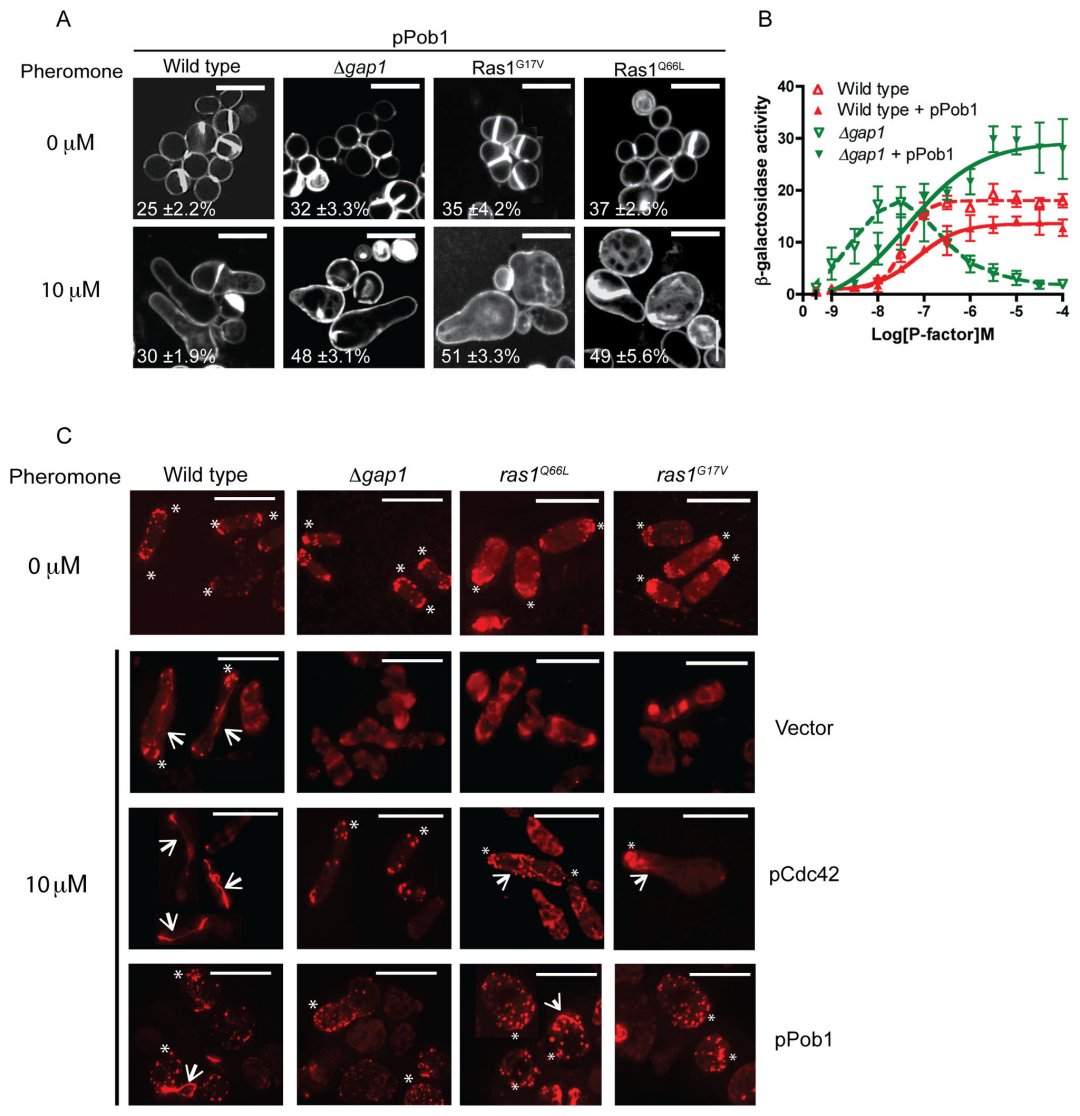
Loss of Ras1-GTP hydrolysis prevents coordination of actin polymerization following pheromone stimulation. **A**, Cells transformed with Pob1 (pPob1) were treated with pheromone and imaged following staining with calcofluor white. Values shown are percentage loss of cell viability. **B**, Pheromone-dependent reporter gene activity was quantified using the *sxa2>lacZ* reporter construct in strains expressing pPob1. Pob1 expression enabled an increased transcriptional response in strains lacking *gap1*. All values mean of triplicate determinations (±S.E.M). **C**, Rhodamine-phalloidin staining of actin wild type and strains containing indicated mutations. All mitotically growing cells (0 μM pheromone) display polarized actin at the cell tips (asterix). Following treatment with 10 μM pheromone elongating wild type cells exhibit defined actin patches (asterix) and cables (arrows). All mutant strains failed to coordinate actin polymerization and a single growth site was not defined. These defects were restored upon increased expression of pCdc42 or pPob1. Scale bar 10 μm.

Defects observed in strains containing a temperature sensitive Cdc42 allele have previously been restored through increased expression of the Boi protein homologue, Pob1 [34]. Pob1 is a downstream effector of Cdc42 required for the coordination of actin organization [35]. Overexpression of Pob1 in our mutant strains reduced the pheromone-induced cell death and prevented the occurrence of multiple growth tips (Figure 8A and Table S2). Furthermore, addition of Pob1 enabled strains to generate an elevated Byr2-MAPK-mediated reporter gene transcriptional response when compared to wild type cells (Figure 8B and Figure S2). These results further demonstrate that hyperactivation of Ras1 enables increased MAPK signaling when cell death is prevented. 

Activation of Pob1 is required to enable coordination of actin polymerization [34]. We therefore investigated the role of Ras1-GTP hydrolysis on pheromone-directed actin polymerization. Following pheromone stimulation, polarized actin patches and cables were observed in wild type cells to direct shmoo formation. Hyperactive Ras1 mutant cells failed to display similar polarization of actin. These defects were corrected upon increased expression of either Cdc42 or Pob1 (Figure 8C) suggesting that reduced Ras1-GTP hydrolysis results in inefficient signal transduction via Cdc42 and Pob1 to mediate actin organization resulting in loss of polarity and ultimately cell death. 

**Figure 9:**
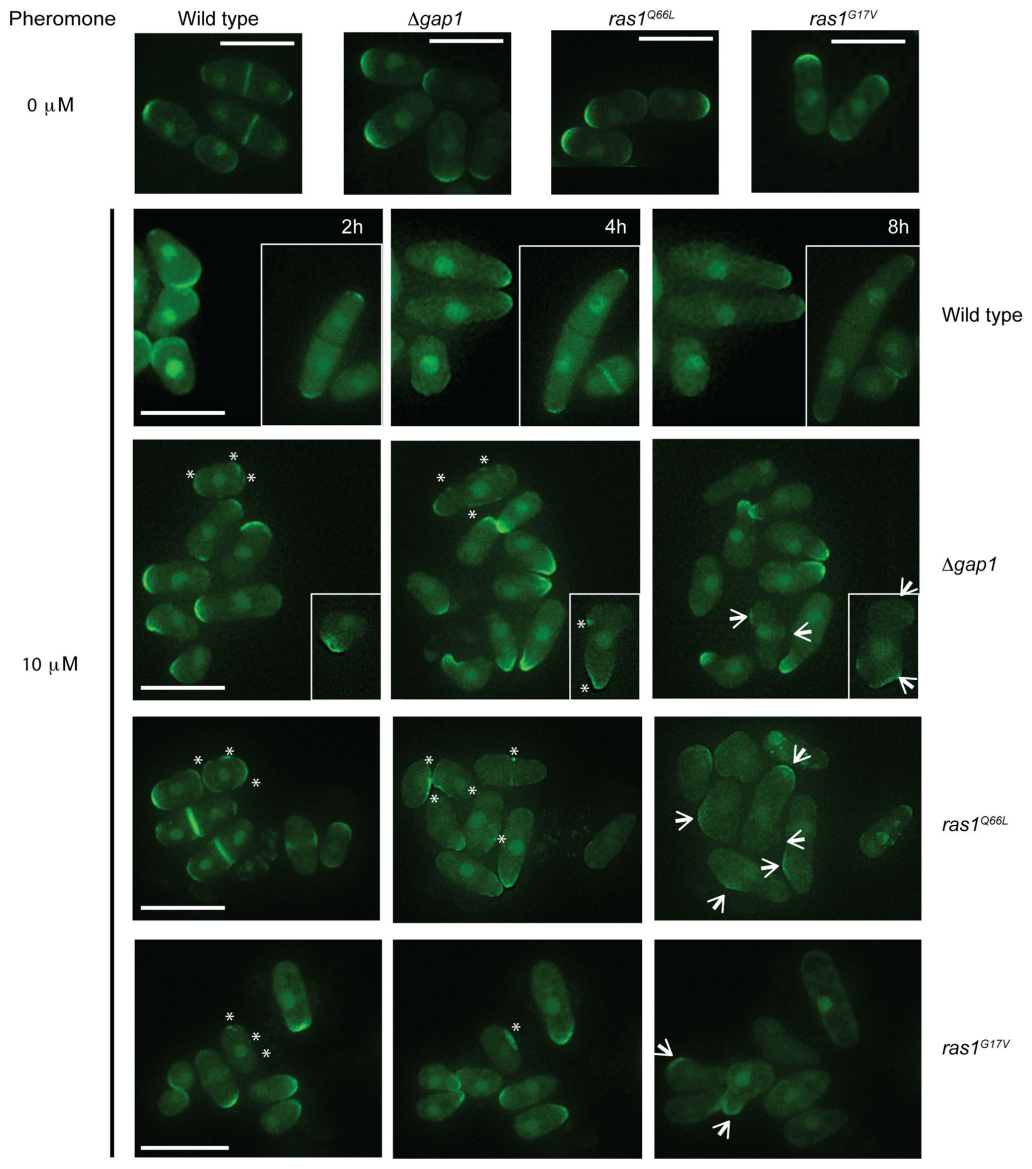
Hyperactivation of Ras1 deregulates Cdc42-GTP localization following pheromone stimulation. CRIB-GFP, a marker for Cdc42-GTP imaged following pheromone stimulation in indicated genotypes, representative images from Movies S5-8. CRIB-GFP is enriched at growing tips in all mitotically growing cells (0 μM). Following 2 h pheromone stimulation CRIB-GFP is enriched at a single tip in wild type cells enabling uni-directional growth to form a polarized conjugation tube. Cells containing hyperactivating Ras1 mutations display CRIB-GFP fluorescence at many discreet locations around the plasma membrane (asterix). These strains fail to coordinate a single site of active Cdc42 and attempt to elongate from multiple sites (arrows). Scale bar 10 μm.

Recently, the importance of Cdc42-GTP in the coordination of fission yeast growth sites during mitotic growth has been demonstrated [5]. It is thought that following pheromone stimulation; an active Cdc42-GTP complex directs actin polymerization at a growth site allowing the accumulation of more Cdc42 in a feed-forward mechanism to establish a single site for elongation. To investigate Cdc42 activity in our mutant strains which display an inability to coordinate actin polymerization and establish unidirectional growth we expressed a GFP-tagged Cdc42/Rac-interactive binding (CRIB) domain, a marker for active Cdc42 [36,37]. In mitotically growing cells, CRIB-GFP localized to the poles of all strains (Figure 9) and was coordinated to the single, growing tip in pheromone-stimulated, wild type cells (Figure 9 and Movie S5). In contrast, cells with reduced Gap1-mediated Ras1-GTP hydrolysis displayed less polarized CRIB-GFP localization instead accumulating as discrete spots around the cell membrane (Figure 9, asterix). Recently, Bendezú and Martin described how, upon initial detection of pheromone, an active Cdc42 complex appears to scan the periphery of the cell. Prolonged exposure results in accumulation of active Cdc42 to a single site initiating shmoo formation [6]. Time-lapse imaging of our strains expressing CRIB-GFP (Movies S5-8) suggests that cells lacking negative regulation of Ras1 initiate the Cdc42 periphery scanning process (Figure 9, asterix) but are unable to coordinate a single growth zone upon prolonged pheromone exposure presumably through an inability to polymerize actin cables (Figure 8). Consequently these cells, which have lost their ability to polarize growth, initiate shmoo formation from multiple sites (Figure 9, arrows) but fail to sustain the response and ultimately die. These data are the first to demonstrate the importance of Ras1-GTP hydrolysis for efficient signal transduction via Cdc42 to establish and maintain cell polarity during growth. 

## Discussion

### Gap1 interaction with Ras1-GTP

How cells regulate their shape is a fundamental question in biology. Many specialized cells, for example migratory T-cells and neurons, have specific functions that require them to maintain a defined polarity in order to respond appropriately to external signals. The small GTPases such as Ras and Cdc42 perform essential roles in this process. Recently, there have been several studies using the fission yeast, *S. pombe* surrounding the importance of Cdc42 regulation during polarized cell growth [5,6]. Although, analogous to the role of Ras in other systems, Ras1 is known to be required to regulate fission yeast cell morphology, there are still many questions regarding its regulation [17,38]. In this study we have investigated the importance of Ras1-GTP hydrolysis in the regulation of polarized cell growth as summarized in Table 1. Initially we confirmed that Gap1, a predicted Ras1-GAP [13], interacts with (active) Ras1-GTP. Through development of a FACS-FRET assay we quantified the dynamics of this interaction. We next used the FACS-FRET technique to investigate GAP-binding for two hyperactivating mutations, Ras1^G17V^ and Ras1^Q66L^. We demonstrated that, analogous to mammalian cells [21], mutation of the second glycine (GD**G**GVGKS – residue highlighted in bold) to valine within the P-loop of Ras1 prevents GAP-binding. In contrast, mutation of the critical glutamine residue (required to orientate the catalytic water molecule during GTP hydrolysis) [39] to leucine in the switch II domain did not prevent Gap1-binding, however GTP hydrolysis was impaired (detected as a prolonged FRET signal). Intriguingly, this transition appeared to result in constitutive GAP-binding (observed as colocalization at the plasma membrane in the absence of pheromone stimulation). This data suggests that Ras1^Q66L^ adopts an active conformation since GAPs selectively bind to the activated state of G proteins [40] however, pheromone stimulation was required for effector activation (Figure 4B) and the production of a FRET signal (Figure 3E). Taken together we hypothesize that the Q66L transition produces a conformation with a high affinity for Gap1-binding. However, pheromone stimulation is required to induce a structural change (detected as a FRET signal) to enable activation of downstream effectors. Since GAPs cannot bind the GDP-bound state of G proteins these observations lead to an interesting hypothesis whereby Ras1^Q66L^ adopts an intermediary conformation that is neither GDP- (facilitating interaction with Gap1) nor GTP-bound (preventing activation of Byr2).

**Table 1:** Summary of pheromone-induced defects in Ras1-mediated signaling.

		10 μM Pheromone			
Strains		Cell Death	MAPK	Shmoo	Mating
Wild type	Strain alone	**-**	**++**	**++**	**++**
	*Δbyr2*	**-**	**-**	**-**	**-**
	*Δscd1*	**-**	**++**	**-**	**-**
	+ pRga4	**-**	**++**	**++**	**++**
	+ pCdc42	**-**	**+**	**++**	**+**
	+ pScd1	**++**	**+**	**+**	**-**
	+ pPob1	**+**	**++**	**++**	**++**
Hyperactive Ras1 mutants	Strain alone	**+++**	**-**	**-**	**-**
	*Δbyr2*	**-**	**-**	**-**	**-**
	*Δscd1*	**-**	**++**	**-**	**-**
	+ pRga4	**+++**	**-**	**-**	**-**
	+ pCdc42	**++**	**+**	**++**	**+**
	+ pScd1	**+++**	**-**	**-**	**-**
	+ pPob1	**+**	**+++**	**++**	**++**

Data has been collated from the assays previously described and conducted in this study. Hyperactive Ras1 mutants include fission yeast strains lacking *gap1* or containing either Ras1^G17V^ or Ras1^Q66L^. The extent of the response is represented as follows: **+++** increased level of response, **++**normal level of response, **+** reduced level of response and - no response.

### Ras1-GTP hydrolysis is essential for coordination of Cdc42-GTP

We have shown that upon hyperactivation of Ras1, fission yeast cells fail to coordinate active Cdc42 to establish a single growth zone following pheromone stimulation, instead promoting disorganized actin deposition. *S. pombe* has a rod-like shape that is regulated by the cell cycle and external cues such as mating pheromones. To maintain this polarity, the precise spatial and temporal regulation of the small GTPases, Cdc42 and Ras1 are essential [3]. Cdc42 regulates cytoskeletal asymmetry and is activated by two GEFs, Gef1 and the Ras1-activated Scd1 such that deletion of Scd1 or Ras1 generates round, non-polar cells. Polarization of the actin cytoskeleton, through localization of active Cdc42, recruits Scd1. Therefore; Cdc42 activity is self-regulated in a feed-forward mechanism, enabling cell growth. During mitotic growth, immediately after cell division, Cdc42 activity is directed to the tip that existed before cytokinesis (the old end). At a defined time within the cell cycle (attainment of a minimal cell size) [41] Gef1 activity mediates the transition to bipolar growth with active Cdc42 oscillating between both tips [5]. Upon pheromone stimulation, the Ras1-Scd1 pathway directs Cdc42-GTP to a single tip enabling orientation and cell elongation towards a mating partner (Figure 9). 

Recently it has been demonstrated that on initial detection of pheromone, an active Cdc42 complex appears to scan the periphery of the cell. Following prolonged exposure, a single site is specified for growth with Cdc42-GTP accumulating in a mechanism similar to that described for mitotic growth [6]. We suggest that in the absence of Ras1-GTP hydrolysis, cells initiate the membrane exploration phase but Cdc42-GTP is unable to interact with downstream effectors to polarize actin and recruit sufficient activity to define the single, ‘winning’ site. These cells appear to accumulate uncoordinated pockets of active Cdc42 around the cell periphery that persist and appear resistant to negative regulation by Rga4 (the Cdc42 GAP). The resultant ectopic accumulation of Cdc42-GTP initiates growth at multiple sites, which is unsustainable for the cell. These pockets appear to sequester Cdc42 and its activator Scd1, thereby preventing it from performing essential roles in mediating *routine* cellular processes leading to loss in cell viability. We propose that, in the absence of Ras1-GTP hydrolysis, Cdc42-GTP is retained within the Ras1-GTP-Scd1-Cdc42-GTP complex, thereby inhibiting interaction with its essential downstream effectors and preventing actin organization. These data reveal an essential requirement for Ras1-mediated GTP hydrolysis to transduce signals via Cdc42. 

### Ras1-GTP hydrolysis differentially regulates signal transduction

Our data also highlights a striking contrast in the mechanisms used by downstream effectors to propagate a response. As in higher eukaryotes, Ras1 interacts with multiple effectors, including Scd1 for actin cytoskeleton remodeling and Byr2, for MAPK-mediated gene transcription. When strains expressing the activating Ras1 mutations were prevented from dying (through treatment with a lower concentration of pheromone or deletion of Scd1) an elevated transcriptional response, compared to strains containing a wild type Ras1 protein, was observed. This data is consistent with reports of oncogenic Ras mutants (deficient in GTPase activity) promoting unregulated cell proliferation via classical growth factor signaling cascades [42]. These observations suggest that the downstream effectors of Ras1 differentially require GTP hydrolysis to propagate a response and could be highly significant when we consider the vast array of signal transduction pathways regulated by Ras proteins in mammalian cells.

 The proteins involved in the regulation of cell polarity are highly conserved among eukaryotic cells; for example, Cdc42 regulates actin cytoskeleton remodeling for T-cell migration and neuronal growth. Similarly, in mammalian cells, Ras proteins regulate both MAPK cascades for gene transcription and Rho-GTPases (such as Cdc42) to maintain polarity. Activating mutations in Ras proteins have been associated with tumor formation. However, whilst much research into their affects on MAPK signaling has been undertaken [43], little appears to be known about oncogenic transitions and cell polarity. The observations we describe, that Ras1-GTP hydrolysis may prevent excessive Cdc42-mediated actin organizing, are of considerable interest given the many diseases surrounding Ras-mediated, uncontrolled cell growth and migration.

## Materials and Methods

### Strains, reagents and general methods

Yeast strains used in this study are listed in Table S3. With the exception of JY444 and JY1025, all yeast strains were derived from JY546 which contains the *sxa2>lacZ* construct for quantification of pheromone-dependent transcription [23,44]. The strains are also deleted for *cyr1* to induce sexual differentiation. Deletion of *cyr1* results in strains undergoing cell division at a smaller size although they maintain their characteristic barrel morphology [15]. Generation of Ras1 mutants integrated at the *ras1* locus was performed using a two-step gene replacement strategy. The endogenous *ras1* was deleted using the *ura4* cassette, which was subsequently replaced by either Ras1^G17V^ or Ras1^Q66L^. *scd1* and *byr2* were single-step deletions using the *kan* ^*R*^ cassette. Deletion of Mam2 has been described previously [24,44]. To enable consistent comparison with the parent strains, which were *ura4* ^*-*^, the Ura4 ORF was subsequently deleted from the locus of strains using a *Bam*HI digest of JD437 (pKS^+^ Bluescript containing the 5’ and 3’ un-translated regions of the *ura4* cassette separated by an *Eco*RV site) and selection by growth on minimal media plates containing 5-fluoro-ortic acid (5-FOA). We have used similar techniques for disruption of other members of the *S. pombe* pheromone-response pathway [44]. To produce strains expressing the CRIB-3xGFP reporter construct we generated strains (JY944, JY1272, JY1386 and JY1538) that contained randomly generated point mutations within the Ura4 cassette (contained downstream of Krp1) [23], which were selected by growth on minimal media plates containing 5-FOA. The *shk1* promoter:ScGIC2 CRIB domain:3xGFP (a gift from Kaz Shiozaki) was then integrated by selection on minimal media plates lacking uracil. Colonies were screened by microscopy to select successful recombinants expressing CRIB-GFP. Gene replacements were confirmed using polymerase chain reaction (PCR). General yeast procedures were performed as described previously [16,25] using yeast extract (YE) media for routine cell growth and selective, defined minimal media (minimal media) for all assays. Our minimal growth media is a variant of EMM as described by Davey et al. [16], and places a higher nutritional demand on the cell resulting in the cells undergoing division at a smaller size [14]. For mild-overexpression of Cdc42, cells containing pREP4x-Cdc42 were grown in minimal media with the addition 5 μg/ml thiamine. Oligonucleotides were synthesized by Invitrogen (Paisley, Scotland, UK) amplification by PCR used FastStart high fidelity polymerase blend (Roche Diagnostics Ltd., UK). 

### Plasmids

The pREP vectors allow expression of genes under the control of the thiamine-repressible, *nmt1* promoter; pREP3x contains the nutritional selection marker *LEU2* and pREP4x, *ura4* [45]. To account for differences in plasmid expression levels between individual cells, all yeast strains containing plasmids were grown for 48 hours in the absence of thiamine to ensure full expression from the *nmt1* promoter was achieved [45] before assays and imaging were performed. *S. pombe* genes were amplified from genomic DNA using PCR and cloned into the pREP vectors. All Ras1 mutations were generated through inverse PCR and constructs sequenced to ensure faithful amplification. Fluorescent constructs were generated using a two-step cloning technique as described previously [24]. CFP was amplified using the sense primer JO2518 (ATGGTGAGCAAGGGCGAGGAG) and antisense primer JO2590 ( atcCTTGTACAGCTCGTCCATGCCG) which includes half of an *Eco*RV site (underlined) in place of the stop anticodon. The PCR product was cloned into the unique *Eco*RV site of a modified pREP vector to generate JD3520 (pREP4x-CFP) thereby also re-creating the *Eco*RV site in place of stop anticodon. All Ras1 constructs were amplified using JO1953 (ATGAGGTCTACCTACTTAAGAGAG) and JO1925 (CTAACATATAACACAACA) containing the stop anticodon (shown in italics) and were cloned into the *Eco*RV site of JD3520. The additional nucleotides (gat) inserted between the *ras1* ORF and the CFP ORF introduces an aspartate residue. YFP was amplified using the sense primer JD2318 ( atcATGGTGAGAAAGGGCGAGGAG) which includes half of an *Eco*RV site (underlined) and JO2319 (TTACTTGTACAGCTCGTCCATGCCG) containing the stop anticodon (shown in italics). This PCR product was cloned into the unique *Eco*RV site of a modified pREP vector to generate JD3521 (pREP3x-YFP) thereby also re-creating the *Eco*RV site immediately upstream of the initiator codon of YFP. Gap1 was amplified using JO2764 (ATGACTAAGCGGCACTCTGG) and JO2828 (CTTTCGTAAAAACAATTGTTCAAATAAAT) removing the stop anticodon from the ORF and were cloned into the *Eco*RV site of JD3521. The additional nucleotides (atc) inserted between the *gap1* ORF and the YFP ORF introduces an isoleucine residue. 

### β-galactosidase assay

Assay of β-galactosidase activity was performed as previously detailed [23,46]. Activity is displayed as OD_420_ per 10^6^ cells. Cell number and median cell volume were determined using Z_2_ Coulter channelyzer (Beckman Coulter, Luton, UK). 

### Mating assay

To determine mating efficiency we used a quantitative spore viability assay. Many of the strains used are Sxa2 null mutants and are sterile [13,26]. To enable mating assays to be performed, we generated h^-^ strains lacking Gap1 (JY1641) or expressing the hyperactivating Ras1 mutations (Ras1^G17V^ (JY1642) or Ras1^Q66L^ (JY1643)) from JY444 (h^-^, *cyr1* ^*-*^) [47]. To quantify spore viability yeast cultures were grown to a density of ~ 5 x10^6^ cells/ml in minimal media and 200 μl of each test strain was mixed with JY1025 (h^+^, *cyr1* ^*-*^) [48]. Cells were harvested at 2000 rpm for 3 min, the pellet was re-suspended in 10 μl of sterile water and spotted on to a minimal media plate with 1 in 100 dilution of nitrogen. After 72 h incubation at 29°C each spot was picked into 1 ml of water. 500 μl of this culture was taken and vegetatively cells were heat inactivated at 55°C for 10 min. Heated and unheated samples were plated on to yeast extract plates and incubated for a further 48 h at 29°C to allow spores to form colonies. Spore viability was determined as:

*% recovery = colonies formed after heat treatment / colonies formed with no heating*

### Flow cytometry

Flow cytometry was performed using a Beckton, Dickinson and Company (BD) LSRII flow cytometer (BD Biosciences, Oxford, UK). Cell cycle analysis using propidium iodide staining utilized an adapted protocol from Sazer and Sherwood, [49]. Following overnight fixation with 70% ethanol, cells were washed in 50 mM sodium citrate and digested for 2 h with 0.1 mg/ml RNase. DNA was stained with 4 μg/ml propidium iodide and quantified using excitation with 488 nm laser detected with 575/26 nm bandpass filter and 550 nm long pass. CFP fluorescence was detected after excitation with 405 nm laser with 450/50 nm filter and YFP excitation was achieved with a 488 nm laser and emission taken by 530/50 nm filter (505 nm long pass). To determine FRET we excited with 405 nm laser and collected emission with 585/42 nm filter set and 545 nm long pass. Gating to determine cells displaying a FRET signal is shown in Figure S1F and described previously [50]. Cell viability was calculated after pheromone treatment using the LIVE/DEAD^®^ *Funga* light^TM^ yeast viability kit (Invitrogen Ltd) according to manufacturers protocol. Briefly, cells were treated with varying concentrations of pheromone, washed three times in phosphate buffered saline and 0.5 μl of each viability dye reagent (SYTO 9 and propidium iodide) was added. The proportion of cells containing both dyes was reported as percentage of cells containing SYTO 9. All analysis was performed using FACSDiva v4.1 (BD Biosciences).

### Microscopy

Bright field microscopy time course experiments were performed on 2% minimal media agarose plugs at 29°C with images taken every 15 min. Bright field microscopy and fluorescent images were taken using a True Confocal Scanner Leica TCS SP5 microscope (Leica Microsystems Ltd., Milton Keynes, UK). Actin patches and cables were stained using rhodamine-conjugated phalloidin (Universal Biologicals) via a protocol adapted from Marks et al., [51]. Briefly, 1 ml cells were fixed for 10 min with the addition of 100 μl 37% formaldehyde. Fixed cells were washed 3 times in PEM (0.1 M Na PIPES pH 6.8, 1 mM EGTA, 1 mM MgCl_2_) and extracted for 30 sec in PEM + 1% TritonX-100. 100 μl fixed and extracted cells were harvested and stained overnight at 4°C with 7 μl phalloidin (6.6 μM stock). Actin was visualized using a Personal DeltaVision (Applied Precision, Issaquah, WA) comprising, an Olympus UPlanSApo 100x, N.A. 1.4, oil immersion objective and a Photometric CoolSNAP HQ camera (Roper Scientific). Captured images were processed by iterative constrained deconvolution using SoftWoRx (Applied Precession) and analyzed using ImageJ. To visualize cell wall and septa, cells were harvested and re-suspended in 0.1 mg/ml calcofluor white (Sigma) and imaged using the Leica TCS SP5 microscope (Leica Microsystems Ltd, UK). For the CRIB-GFP, images were taken every 10 minutes using the Personal DeltaVision and the image in the z-stack with the highest intensity chosen.

### FRET measurements using confocal microscope

In all FRET experiments, cells with medium fluorescence of CFP and YFP fusion constructs were selected. We used the sensitized emission method for FRET measurements with detection of fluorescence from single donor (CFP-Ras1), FRET, and acceptor (Gap1-YFP) in a line by line sequential scan acquisition. To correct for excitation and emission crosstalk, calibration coefficients were calculated with the donor-only and acceptor-only references. Parameters were first adjusted with cells expressing the positive (CFP-YFP fusion) and negative (pCFP and pYFP) controls (Figure S1E) and remained constant for the entire experiment. The calibration factor β corrects for donor crosstalk and was calculated from the donor only reference by dividing the CFP donor emission (excited 458 nm laser) in the YFP channel by the CFP donor emission (excited 458 nm laser) in the CFP channel. Similarly, the calibration factor γ corrects for acceptor cross-excitation and was calculated from the acceptor only reference by dividing the YFP channel emission excited by the donor (excited 458 nm laser) with the YFP channel emission of YFP (excited 514 nm laser). Once all calibration factors have been calculated, a region of interest (around a single cell) for the FRET measurement was determined in the FRET image and was automatically inserted by the software at the identical position in the donor and acceptor channels. The apparent FRET efficiency (EA) was calculated by the equation: Ea (i) = B − A * β – C * γ/C [52]. FRET analysis was performed using the Leica Application Suite, Advanced Fluorescence 1.8.0 software. In all experiments, CFP was excited by a 458 nm laser line and detected at 465 to 500 nm. YFP was excited by a 514 nm laser line and detected at 524 nm to 600 nm.

### Data analysis

Data were analyzed using linear and non-linear regression as appropriate using GraphPad Prism v6.0b for Mac OS X (GraphPad Software Inc, San Diego, CA). Statistical significance was determined using a one-way ANOVA with a Tukey multiple comparisons post-test or an un-paired Student’s t test as appropriate. FRET and colocalization analysis was performed using ImageJ 1.46b [53,54].

## Supporting Information

Figure S1 **Measuring CFP-YFP FRET by FACS.** **A**, *Δras1* strains transformed with either Ras1 or a CFP-Ras1 fusion constructs were imaged. *Δras1* (unlike wild type cells) have a rounded morphology that is complemented upon expression of plasmid-borne Ras1. Scale bar = 10 μm B, Mating efficiency was determined using a spore viability assay for h^-^ cells deleted for Ras1 and expressing either empty vector or plasmid borne Ras1 or CFP-Ras1. Both wild type Ras1 and the CFP-fusion were able to restore wild type mating to the *Δras1* cells when expressed from a plasmid. **C**-**D**, *Δgap1* strains transformed with Gap1-YFP were assayed for pheromone-induced changes in gene transcription C, and cell death D. Expression of Gap1-YFP restored signal transduction to near wild type levels. **E**, Imaging using the FRET-sensitization emission method for cells expressing CFP and YFP (negative control) or a CFP-YFP fusion construct (positive control) from thiamine-inducible plasmids. Images were analyzed for colocalization and FRET (see Methods). Cells expressing CFP and YFP from individual plasmids gave a high degree of colocalization (r = 0.9) but this did not translate to a significant FRET signal. In contrast the CFP-YFP fusion construct displayed both a high colocalization (r = 0.9) and a FRET signal. **F**, Gating strategy to measure FRET by FACS (see Methods). Cells containing CFP, YFP, CFP and YFP and a CFP-YFP fusion expressed from plasmids were analyzed as described in the methods section. Gates were set to select double positive cells (panel 1), remove false positive FRET signals (panel 2) and define a positive (red) FRET signal (using CFP and YFP co-transfected population in panel 3).
(TIF)

Figure S2 **Increased expression of Pob1 restores gene transcription on Ras1-GTPase defective strains.** **A**, Cells containing the Ras1^G17V^ and **B**, Ras1^Q66L^ mutation were transformed with various plasmids (see labels) and assayed for pheromone-dependent transcriptional response using the *sxa2>lacZ* reporter. All data are mean of triplicate determinations (±SEM).
(TIF)

Movie S1 **Wild type cells were grown on minimal media containing agarose and supplemented with 10 μM pheromone.** Images were obtained every 15 min, complied and exported as movies with a frame rate of 5 frames per second. Wild type cells elongate from a single tip in response to pheromone displaying characteristic shmoos (original data for Figure 4A).
(AVI)

Movie S2 **Cells lacking Gap1 were treated as described for Movie S1.** Many aberrant responses were observed upon pheromone treatment including multiple projection tips, enlarged vacuoles and cell lysis (original data for Figure 4A).
(AVI)

Movie S3 **Cells expressing the Ras1^G17V^ mutant were treated as described for Movie S1.** Similar defects in morphology were observed upon pheromone treatment (including multiple projection tips, enlarged vacuoles and cell lysis) to those displayed for cells lacking Gap1.
(AVI)

Movie S4 **Cells expressing the Ras1^Q66L^ mutant were treated as described for Movie S1.** Similar defects in morphology were observed upon pheromone treatment (including multiple projection tips, enlarged vacuoles and cell lysis) to those displayed for cells lacking Gap1.
(AVI)

Movie S5 **Wild type cells containing CRIB-GFP were grown on minimal media containing agarose and supplemented with 10 μM pheromone.** Images were obtained every 10 min, complied and exported as movies with a frame rate of 5 frames per second. Wild type cells coordinate a single site of active Cdc42 (observed as an accumulation of CRIB-GFP) enabling polarized elongation from a single tip (original data for Figure 8).
(AVI)

Movie S6 **Cells lacking Gap1 and containing CRIB-GFP were treated as described for Movie S5.** In the absence of Gap1 cells fail to coordinate a single site of active Cdc42 instead displaying discreet dots of CRIB-GFP, which appear to scan the cell membrane. Many cells attempt to elongate from multiple sites (original data for Figure 8).
(AVI)

Movie S7 **Cells expressing the Ras1^G17V^ mutation and containing CRIB-GFP were treated as described for Movie S5.** Similar defects were observed upon pheromone treatment to those displayed for cells lacking Gap1 (original data for Figure 8).
(AVI)

Movie S8 **Cells expressing the Ras1^Q66L^ mutation and containing CRIB-GFP were treated as described for Movie S5.** Similar defects were observed upon pheromone treatment to those displayed for cells lacking Gap1 (original data for Figure 8).
(AVI)

Table S1 **Asco-spore viability for strains expressing Ras1-GTP hydrolysis mutations.**
(DOCX)

Table S2 **Summary table of cell viability.** To determine the number of dead cells within a population we utilized a cell viability assay (see Methods). Viability was determined in each case before and after treatment with pheromone and values are the means (±SEM) of triplicate determinations. Statistical significance was determined using a Student’s t test. Differences were considered significant as follows; * *p* < 0.05, ** *p* < 0.01, *** *p* < 0.005 from the same strain expressing vector control.
(DOCX)

Table S3 ***Schizosaccharomyces pombe* strains used in this study.**
(DOCX)
