# Expression of Concern: The Coordination of Cell Growth during Fission Yeast Mating Requires Ras1-GTP Hydrolysis

**DOI:** 10.1371/journal.pone.0223817

**Published:** 2019-10-10

**Authors:** The PLOS ONE Editors

After publication of this article [1], the following concerns were raised:

- In Fig 8A, abrupt discontinuities are visible when the image for the strain Ras1^G17V^ at 0 μM is adjusted for brightness.
- In Fig 8C, abrupt discontinuities are visible in the following panels when adjusted for brightness/contrast. The discontinuities suggest that the image in each panel of concern may not represent continuous data from the same photograph.
  ◦ wild type transformed with vector
  ◦ wild type transformed with pCdc42
  ◦ *Δgap1* transformed with pCdc42
  ◦ *ras1*^*Q66L*^ transformed with vector
  ◦ *ras1*^*Q66L*^ transformed with pPob1
- Similarities were noted between the images in the following panels:
  ◦ Fig 6C *Δgap1Δbyr2*/0 μM, Fig 6C *Δgap1*+pScd1/0 μM, and Fig 7C Ras1^G17V^/0 μM
  ◦ Fig 6C *Δgap1*/0 μM and *Δgap1Δbyr2*/10 μM
  ◦ Fig 6A *Δbyr2*/0 μM (right) and Fig 7C Ras1^Q66L^/0 μM

The authors claimed that Fig 8A and 8C panels each show a composite montage of cell images captured from nonadjacent areas of the same slide, similar to Figure 9. They provided an updated version of Fig 8C in which image splice lines are indicated.

They also noted that errors were made in preparing figures which led to the duplications in Figs 6 and 7. In the published figures, the incorrect images are shown in Fig 6C for *Δgap1*/0 μM and *Δgap1*+pScd1/0 μM; and in Fig 7C for Ras1^G17V^/0 μM and Ras1^Q66L^/0 μM. The authors provided revised figures in which the errant panels in Figs 6 and 7 have been replaced with the correct data from the original experiments.

Please see the updated Figs 6–8 and their accompanying captions here. Note, legends for Figs 6 and 7 are unchanged from the published article.

**Fig 6:**
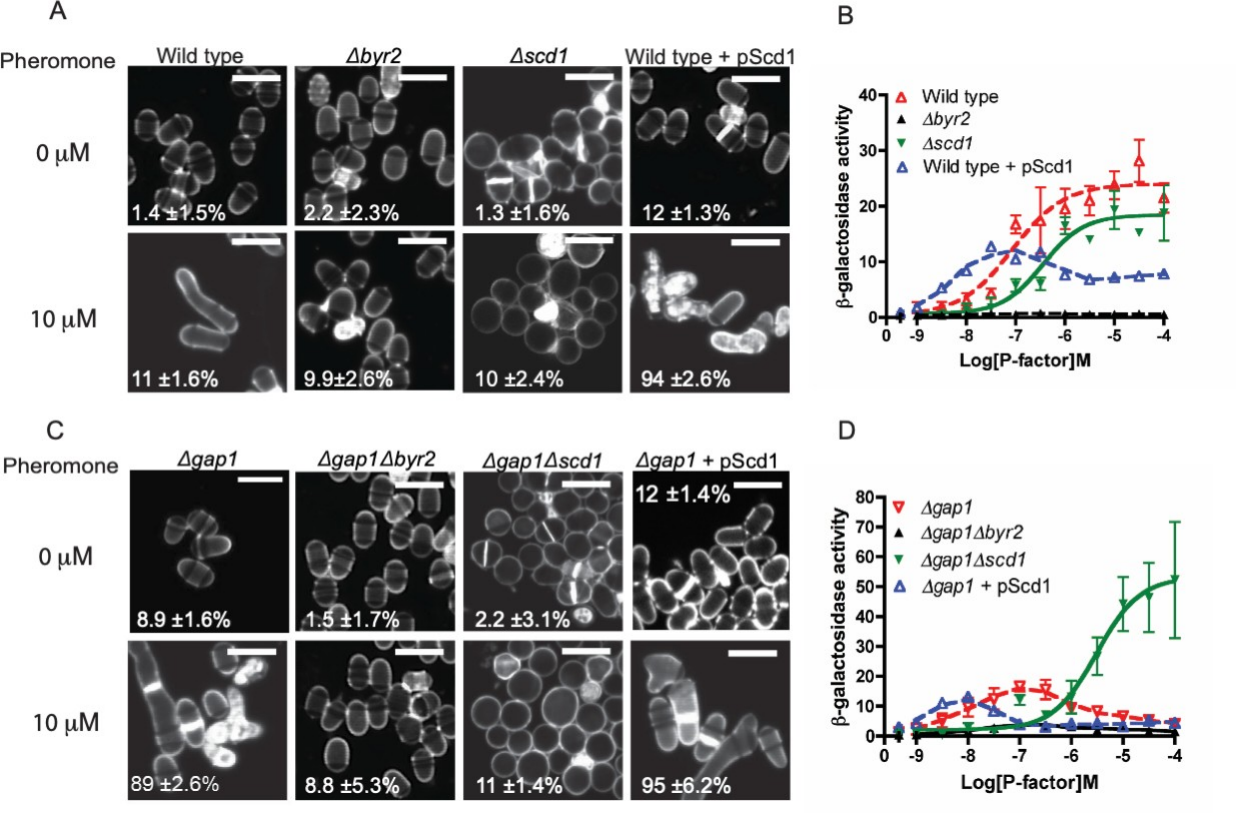
Ras1-GTP hydrolysis is essential to mediate cell polarity but not MAPK signaling. **A** and **C**, Calcofluor white staining of indicated strains treated with 10 μM pheromone. Scale bar 10 μm. Values shown are percentage loss of cell viability for each population. **B** and **D**, Pheromone-dependent transcription as determined using the *sxa2>lacZ* reporter for the stains indicated. Removal of Scd1 from cells lacking Gap1 prevented pheromone-induced cell death while enabling an elevated transcriptional response. All values are mean of triplicate determinations (±SEM).

**Fig 7:**
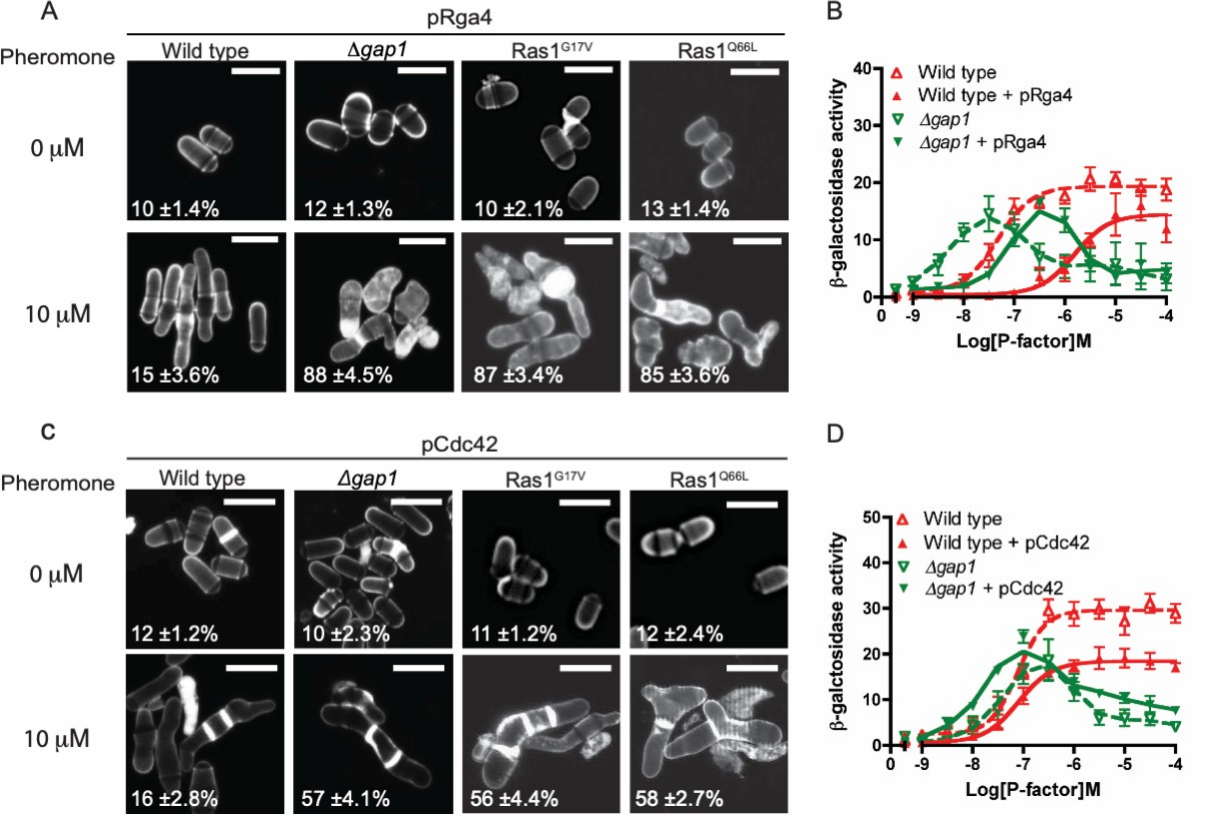
Pheromone-induced cell death is due to reduced Cdc42 signal transduction. **A**, **and C** Values shown are percentage loss of cell viability. Scale bar 10 μm **A**, Calcofluor white staining of cells from indicated genotypes treated with pheromone. **B**, Pheromone-dependent transcription was quantified using the *sxa2>lacZ* reporter construct in strains expressing pRga4. **C**, Cells transformed with Cdc42 (pCdc42) were treated with pheromone, imaged as for **A**, many cells displayed multiple septa (arrows). **D**, Transcriptional response to pheromone was determined and revealed a slight restoration of maximal signal in the *Δgap1* strain containing pCdc42. Values shown are mean of triplicate determinations (± SEM). Scale bar 10 μm.

**Fig 8:**
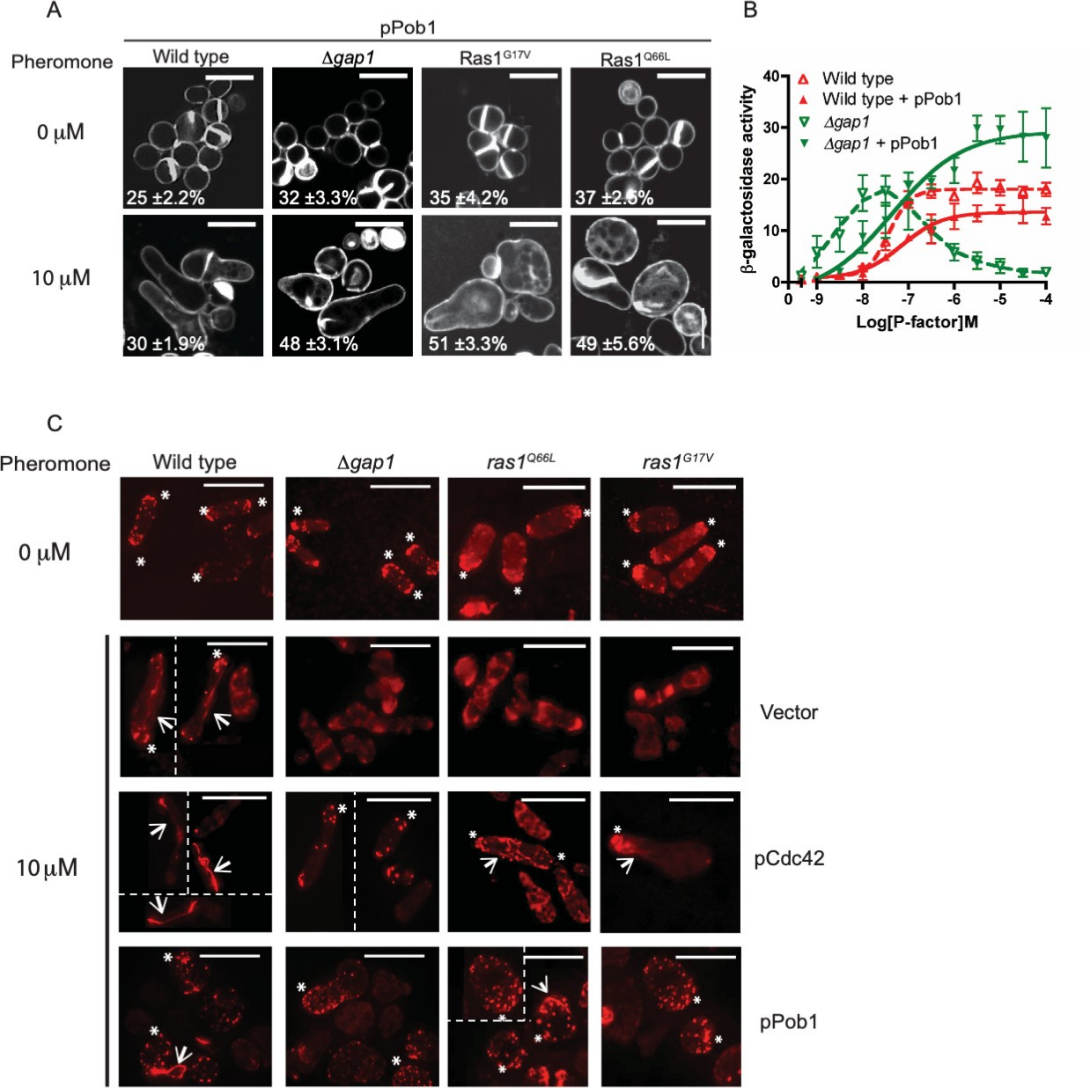
Loss of Ras1-GTP hydrolysis prevents coordination of actin polymerization following pheromone stimulation. **A**, Cells transformed with Pob1 (pPob1) were treated with pheromone and imaged following staining with calcofluor white. Values shown are percentage loss of cell viability. **B**, Pheromone-dependent reporter gene activity was quantified using the *sxa2>lacZ* reporter construct in strains expressing pPob1. Pob1 expression enabled an increased transcriptional response in strains lacking *gap1*. All values mean of triplicate determinations (±S.E.M). **C**, Rhodamine-phalloidin staining of actin wild type and strains containing indicated mutations. All mitotically growing cells (0 μM pheromone) display polarized actin at the cell tips (asterix). Following treatment with 10 μM pheromone elongating wild type cells exhibit defined actin patches (asterix) and cables (arrows). Cells are shown as individuals (where appropriate) and group to form montages. Splice lines (dotted) are added to separate the individual cells so there are no issue with discontinuity. Furthermore, the smear observed for *ras1*^*Q66L*^ transformed with vector is due to the background of the slide when imaging. All mutant strains failed to coordinate actin polymerization and a single growth site was not defined. These defects were restored upon increased expression of pCdc42 or pPob1. Scale bar 10 μm.

The underlying raw image data supporting the figures discussed above are no longer available.

Due to the nature and extent of image issues and lack of underlying image data for the figures in question, the *PLOS ONE* Editors issue this Expression of Concern.
