# Supplementary figures and images for: The Coordination of Cell Growth during Fission Yeast Mating Requires Ras1-GTP Hydrolysis
Source: PLoS One. 2013 Oct 16;8(10):e77487. doi: 10.1371/journal.pone.0077487 (PMC3797800; doi:10.1371/journal.pone.0077487)

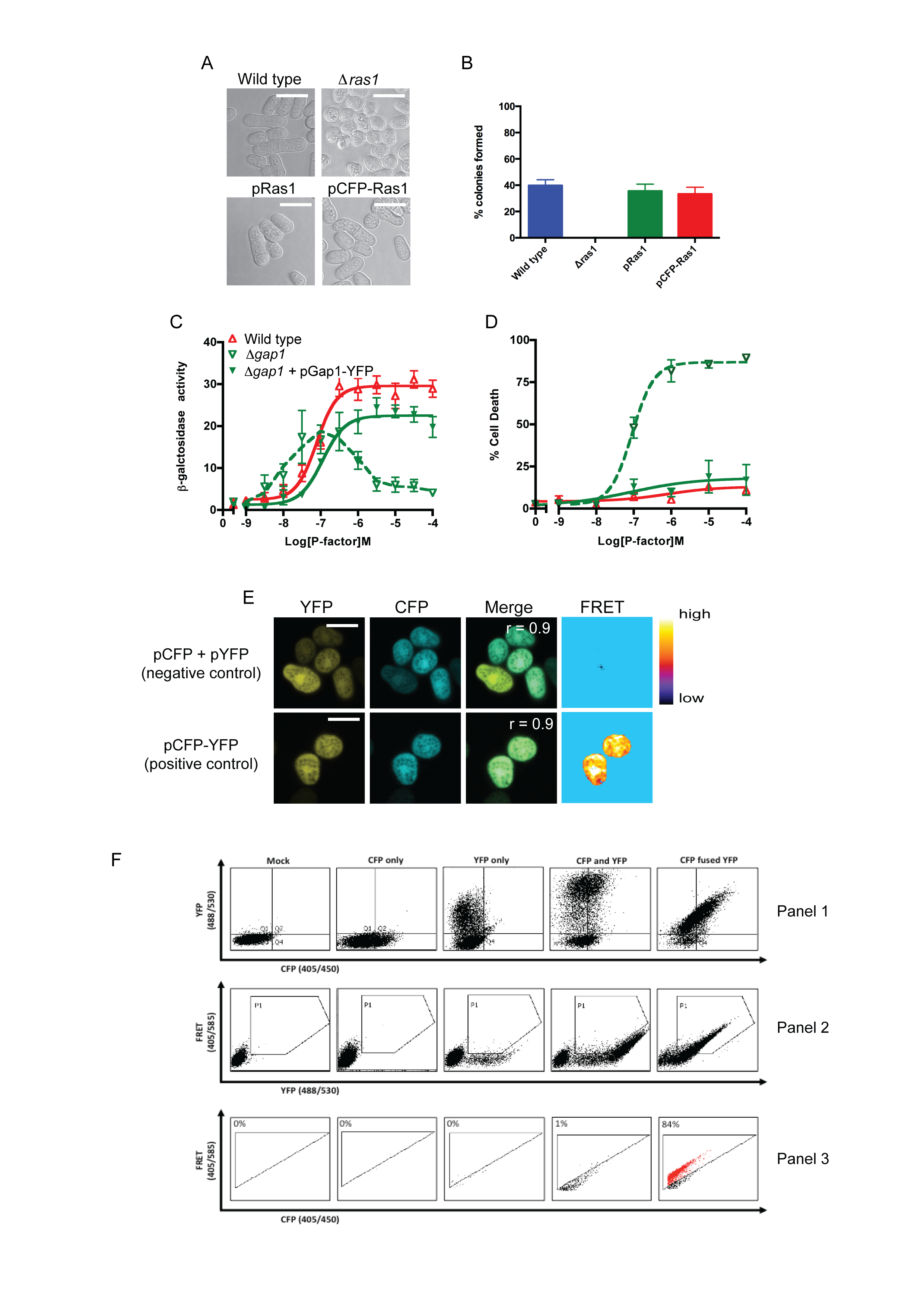

Supplement: Figure S1 — Measuring CFP-YFP FRET by FACS. A, Δras1 strains transformed with either Ras1 or a CFP-Ras1 fusion constructs were imaged. Δras1 (unlike wild type cells) have a rounded morphology that is complemented upon expression of plasmid-borne Ras1. Scale bar = 10 μm B, Mating efficiency was determined using a spore viability assay for h- cells deleted for Ras1 and expressing either empty vector or plasmid borne Ras1 or CFP-Ras1. Both wild type Ras1 and the CFP-fusion were able to restore wild type mating to the Δras1 cells when expressed from a plasmid. C-D, Δgap1 strains transformed with Gap1-YFP were assayed for pheromone-induced changes in gene transcription C, and cell death D. Expression of Gap1-YFP restored signal transduction to near wild type levels. E, Imaging using the FRET-sensitization emission method for cells expressing CFP and YFP (negative control) or a CFP-YFP fusion construct (positive control) from thiamine-inducible plasmids. Images were analyzed for colocalization and FRET (see Methods). Cells expressing CFP and YFP from individual plasmids gave a high degree of colocalization (r = 0.9) but this did not translate to a significant FRET signal. In contrast the CFP-YFP fusion construct displayed both a high colocalization (r = 0.9) and a FRET signal. F, Gating strategy to measure FRET by FACS (see Methods). Cells containing CFP, YFP, CFP and YFP and a CFP-YFP fusion expressed from plasmids were analyzed as described in the methods section. Gates were set to select double positive cells (panel 1), remove false positive FRET signals (panel 2) and define a positive (red) FRET signal (using CFP and YFP co-transfected population in panel 3). (TIF) [file pone.0077487.s001.tif]

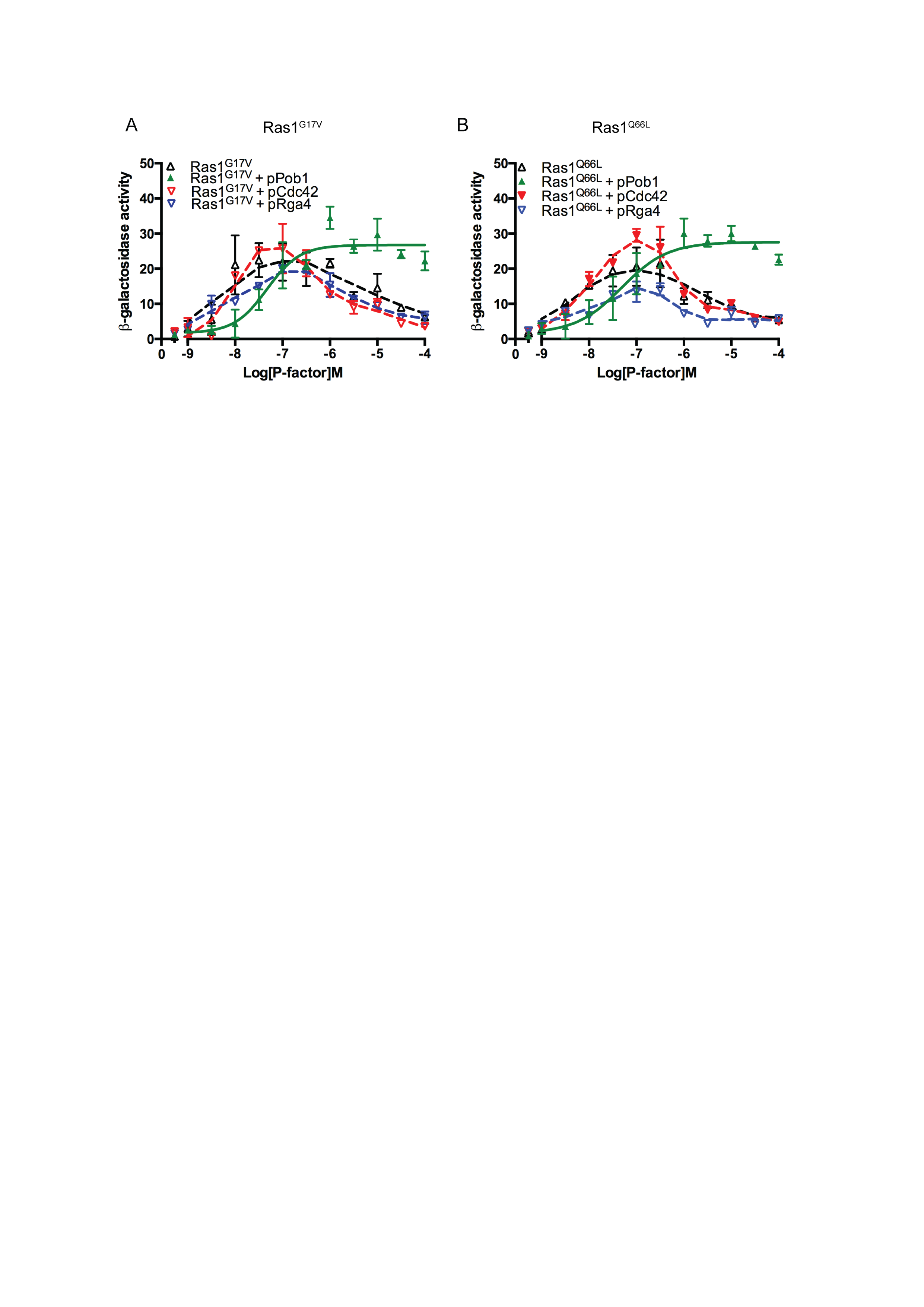

Supplement: Figure S2 — Increased expression of Pob1 restores gene transcription on Ras1-GTPase defective strains. A, Cells containing the Ras1G17V and B, Ras1Q66L mutation were transformed with various plasmids (see labels) and assayed for pheromone-dependent transcriptional response using the sxa2>lacZ reporter. All data are mean of triplicate determinations (±SEM). (TIF) [file pone.0077487.s002.tif]
