# Supplementary material for: The Coordination of Cell Growth during Fission Yeast Mating Requires Ras1-GTP Hydrolysis
Source: PLoS One. 2013 Oct 16;8(10):e77487. doi: 10.1371/journal.pone.0077487 (PMC3797800; doi:10.1371/journal.pone.0077487)
Supplement: Table S1 — Asco-spore viability for strains expressing Ras1-GTP hydrolysis mutations. (DOCX) [file pone.0077487.s011.docx]

| **Plasmid** | **Mating Efficiency (%)** | | | |
| --- | --- | --- | --- | --- |
|  | h^-^ | h^-^ *Δgap1* | h^-^ Ras1^G17V^ | h^-^ Ras1^Q66L^ |
| Vector | 45 ±2.1 | 1.2 ±0.4*** | 1.3 ±0.7*** | 1.1 ±0.8*** |
| pGap1 | 6.0 ±1.1 | 44 ±2.1^***^ | < 0.5 | 2.2 ±0.7 |
| pScd1 | 1.4 ±0.4 | < 0.5 | < 0.5 | < 0.5 |
| pRga4 | 47 ±2.2 | 1.4 ±0.5*** | 2.8 ±0.6*** | 1.9 ±0.3*** |
| pCdc42 | 42 ±1.8 | 18 ±1.9* | 19 ±1.6* | 16 ±3.2* |
| pPob1 | 39 ±2.7 | 32 ±2.0 | 28 ±2.1 | 29 ±2.1 |
