# Supplementary material for: The Coordination of Cell Growth during Fission Yeast Mating Requires Ras1-GTP Hydrolysis
Source: PLoS One. 2013 Oct 16;8(10):e77487. doi: 10.1371/journal.pone.0077487 (PMC3797800; doi:10.1371/journal.pone.0077487)
Supplement: Table S2 — Summary table of cell viability. To determine the number of dead cells within a population we utilized a cell viability assay (see Methods). Viability was determined in each case before and after treatment with pheromone and values are the means (±SEM) of triplicate determinations. Statistical significance was determined using a Student’s t test. Differences were considered significant as follows; * p < 0.05, ** p < 0.01, *** p < 0.005 from the same strain expressing vector control. (DOCX) [file pone.0077487.s012.docx]

|  | | Cell Viability (% cell death) | | | | | |
| --- | --- | --- | --- | --- | --- | --- | --- |
|  |  | Vector | | | | | |
| Strains | P-factor  (μM) | Vector | pGap1 | pScd1 | pRga4 | pCdc42 | pPob1 |
| Wild type | 0 | 11 ±1.2 | 12 ±2.1 | 13 ±1.3 | 10 ±1.4 | 12 ±1.2 | 25 ±2.2 |
|  | 10 | 12 ±2.2 | 13 ±2.4 | 94 ±2.6 | 15 ±3.6 | 16 ±2.8 | 30 ±1.9 |
| *Δgap1* | 0 | 10 ±2.1 | 11 ±1.1 | 12 ±1.4 | 12 ±1.3 | 10 ±2.3 | 32 ±3.3 |
|  | 10 | 88 ±3.6 | 20 ±3.2^***^ | 95 ±6.2 | 88 ±4.5 | 57 ±4.1^*^ | 48 ±3.1^**^ |
| Ras1^G17V^ | 0 | 10 ±2.2 | 11 ±2.2 | 12 ±5.5 | 10 ±2.1 | 11 ±1.2 | 35 ±4.2 |
|  | 10 | 86 ±4.9 | 82 ±2.6 | 89 ±3.9 | 87 ±3.4 | 56 ±4.4^*^ | 51 ±3.3^**^ |
| Ras1^Q66L^ | 0 | 15 ±4.1 | 15 ±1.9 | 15 ±1.6 | 13 ±1.4 | 12 ±2.4 | 37 ±3.5 |
|  | 10 | 85 ±4.3 | 24 ±1.9^***^ | 92 ±5.2 | 85 ±3.6 | 58 ±2.7^*^ | 48 ±5.6^**^ |
