# Supplementary material for: The Coordination of Cell Growth during Fission Yeast Mating Requires Ras1-GTP Hydrolysis
Source: PLoS One. 2013 Oct 16;8(10):e77487. doi: 10.1371/journal.pone.0077487 (PMC3797800; doi:10.1371/journal.pone.0077487)
Supplement: Table S3 — Schizosaccharomyces pombe strains used in this study. (DOCX) [file pone.0077487.s013.docx]

| **Strain** | **Genotype** | **Source** |
| --- | --- | --- |
| JY444 | *mat1-M, Δmat2/3::LEU2^-^, leu1-32, ade6-210, ura4-D18, cyr1-D51.* | [47] |
| JY546 | *mat1-M, Δmat2/3::LEU2^-^, leu1-32, ade6-210, ura4-D18, cyr1-D51, sxa2>lacZ*. | [23] |
| JY994 | *mat1-M, Δmat2/3::LEU2^-^, leu1-32, ade6-210, ura4-D18, cyr1-D51, sxa2>lacZ*, *ura4^+^*. | [23] |
| JY1025 | *mat1-P, ∆mat2/3::LEU2^-^, leu1-32, ade6-210, ura4-D18*. | [48] |
| JY1272 | *mat1-M, ∆mat2/3::LEU2^-^, leu1-32, ade6-210, ura4-D18, cyr1-D51, ras1::ras1^G17V^, sxa2>lacZ*. | This study |
| JY1386 | *mat1-M, Δmat2/3::LEU2^-^, leu1-32, ade6-210, ura4-D18, cyr1-D51, ras1::ras1^Q66L^, sxa2>lacZ*. | This study |
| JY1538 | *mat1-M, Δmat2/3::LEU2^-^, leu1-32, ade6-210, ura4-D18, cyr1-D51, gap1::ura4^-^, sxa2>lacZ*. | This study |
| JY1618 | *mat1-M, Δmat2/3::LEU2^-^, leu1-32, ade6-210, ura4-D18, cyr1-D51, gap1::ura4^-^, ras1::ura4^-^, sxa2>lacZ*. | This study |
| JY1641 | *mat1-M, Δmat2/3::LEU2^-^, leu1-32, ade6-210, ura4-D18, cyr1-D51, gap1::ura4^-^*. | This study |
| JY1642 | *mat1-M, ∆mat2/3::LEU2^-^, leu1-32, ade6-210, ura4-D18, cyr1-D51, ras1::ras1^G17V^*. | This study |
| JY1643 | *mat1-M, Δmat2/3::LEU2^-^, leu1-32, ade6-210, ura4-D18, cyr1-D51, ras1::ras1^Q66L^*. | This study |
| JY1645 | *mat1-M, Δmat2/3::LEU2^-^, leu1-32, ade6-210, ura4-D18, cyr1-D51, sxa2>lacZ*, *ura4^-^*::[shk1 promoter:ScGIC2 CRIB domain:3xGFP:Ura4^+^]. | This study |
| JY1646 | *mat1-M, Δmat2/3::LEU2^-^, leu1-32, ade6-210, ura4-D18, cyr1-D51, gap1::ura4^-^, sxa2>lacZ,* *ura4^-^*::[shk1 promoter:ScGIC2 CRIB domain:3xGFP:Ura4^+^]. | This study. |
| JY1647 | *mat1-M, Δmat2/3::LEU2^-^, leu1-32, ade6-210, ura4-D18, cyr1-D51, ras1::ras1^G17V^, sxa2>lacZ,* *ura4^-^*::[shk1 promoter:ScGIC2 CRIB domain:3xGFP:Ura4^+^]. | This study. |
| JY1648 | *mat1-M, Δmat2/3::LEU2^-^, leu1-32, ade6-210, ura4-D18, cyr1-D51 ras1::ras1^Q66L^, sxa2>lacZ,* *ura4^-^:*:[shk1 promoter:ScGIC2 CRIB domain:3xGFP:Ura4^+^]. | This study. |
| JY1673 | *mat1-M, Δmat2/3::LEU2-, leu1-32, ade6-210, ura4-D18, cyr1-D51, scd1::Kan^R^, sxa2>lacZ.* | This study |
| JY1674 | *mat1-M, Δmat2/3::LEU2-, leu1-32, ade6-210, ura4-D18, cyr1-D51, gap1::ura4^-^, scd1::Kan^R^, sxa2>lacZ.* | This study |
| JY1675 | *mat1-M, ∆mat2/3::LEU2-, leu1-32, ade6-210, ura4-D18, cyr1-D51, gap1::ura4-, byr2::Kan^R^, sxa2>lacZ.* | This study |
| JY1676 | *mat1-M, Δmat2/3::LEU2-, leu1-32, ade6-210, ura4-D18, cyr1-D51, byr2::Kan^R^, sxa2>lacZ.* | This study |
| JY1677 | *mat1-M, Δmat2/3::LEU2-, leu1-32, ade6-210, ura4-D18, cyr1-D51, gap1::ura4^-^, ras1::ura4^-^, Mam2-D10, sxa2>lacZ.* | This study |
